# ATP-citrate lyase B (ACLB) negatively affects cell death and resistance to Verticillium wilt

**DOI:** 10.1186/s12870-022-03834-z

**Published:** 2022-09-16

**Authors:** Fujie Liu, Zhifeng Ma, Sheng Cai, Lingjun Dai, Jianbo Gao, Baoliang Zhou

**Affiliations:** State Key Laboratory of Crop Genetics and Germplasm Enhancement, MOE Cotton Germplasm Enhancement Engineering Research Center, and Collaborative Innovation Center for Modern Crop Production co-sponsored by Province and Ministry, Nanjing Agricultural University, Nanjing, 210095 Jiangsu People’s Republic of China

**Keywords:** ATP-citrate lyase (ACL), Cell death, Cotton, Resistance, *Verticillium dahliae*

## Abstract

**Background:**

ATP-citrate lyase (ACL) plays a pivotal role in histone acetylation and aerobic glycolysis. In plant, ACL is a heteromeric enzyme composed of ACLA (45 kD) and ACLB (65 kD). So far, the function of ACL genes in cotton still remains unknown.

**Results:**

Here, we identified three ACLA homologous sequences and two ACLB homologous in each genome/sub-genome of cotton species. Silencing ACLB in cotton led to cell death at newly-grown leaves and stem apexes. Simultaneously, in ACLB-silenced plants, transcription factors related to senescence including *SGR*, *WRKY23* and *Osl57* were observed to be activated. Further investigation showed that excessive H_2_O_2_ was accumulated, salicylic acid-dependent defense response and pathogenesis-related gene expressions were evidently enhanced in ACLB-silenced plants, implying that knockdown of ACLB genes leads to hypersensitive response-like cell death in cotton seedlings. However, as noted, serious cell death happened in newly-grown leaves and stem apexes in ACLB-silenced plants, which led to the failure of subsequent fungal pathogenicity assays. To confirm the role of ACLB gene in regulating plant immune response, the dicotyledonous model plant *Arabidopsis* was selected for functional verification of ACLB gene. Our results indicate the resistance to *Verticillium dahliae* infection in the *Arabidopsis* mutant *aclb-2* were enhanced without causing strong cell death. Ectopic expression of *GausACLB-2* in *Arabidopsis* weakened its resistance to *V. dahliae* either in Col-0 or in *aclb-2* background, in which the expression level of ACLB is negatively correlated with the resistance to *V. dahliae*.

**Conclusions:**

These results indicate that ACLB has a new function in negatively affecting the induction of plant defense response and cell death in cotton, which provides theoretical guidance for developing cotton varieties with resistance against Verticillium wilt.

**Supplementary Information:**

The online version contains supplementary material available at 10.1186/s12870-022-03834-z.

## Background

Eukaryotes and prokaryotes have a basic biological cell process called programmed cell death (PCD), which controls cell suicide in a conservative and genetically regulated manner, leading to the death of single cells, specific tissues or entire organs [1–3]. In plants, cell death is also precisely regulated. Based on the similarity of cell and biochemical markers to animal cell death, plant PCD is divided into apoptotic cell death, senescent cell death and vacuolar cell death [2, 4, 5]. PCD is not only used as a part of the normal development process, it is also used to cope with the challenge of pathogens.

The innate immune system of plants includes two types: pathogen-associated molecular pattern (PAMP)-triggered immunity (PTI) and effector-triggered immunity (ETI) [6, 7]. One of the most visible manifestations of ETI is hypersensitive responses (HR), in which rapid localized cell death develops at the point of pathogen penetration [8]. As a faster and stronger version of PTI, ETI triggers a series of defense responses to prevent pathogen infection [9, 10]. The Nucleotide-binding leucine-rich repeat (NLR) proteins are intracellular receptors that recognize specific pathogen effector and initiate ETI [8]. The NLR protein functions have only recently been elucidated via structural characterization of HOPZ-ACTIVATED RESISTANCE 1 (ZAR1) in plant [11]. The ZAR1 interacts with HOPZ-ETI-DEFICIENT 1 (ZED1)-related kinases (ZRKs) and AVRPPHB SUSCEPTIBLE 1-like proteins to form a wheel-like pentameric resistosome. The N-terminal α helices (α1) of the five CC domains forms a funnel-shaped structure that triggers HR cell death by translocating into the plasma membrane [12]. Moreover, AtZAR1 resistosome acts as a calcium-permeable channel, triggering immune signaling by increasing cytosolic Ca^2+^ concentrations [13]. A most recent study showed that ZAR1 emerged through gene duplication and that ZRKs were derived from the cell-surface immune receptors wall-associated protein kinases (WAKs) [14].

In plants, there are lesion mimics mutants (LMMs) that spontaneously induce cell death phenotypes. Moreover, random lesions generate on LMMs to activate the overall immune response of the plant, thereby enhance the resistance to diseases [15]. Many LMMs have been reported in a variety of plants, including the model plant *Arabidopsis thaliana* [16, 17], cultivated crops maize [18, 19], barley [20], wheat [21, 22] and rice [23, 24]. More than 40 genes controlling the lesions mimic phenotype have been isolated from LMMs [24], which encode proteins with different functions, such as zinc-finger protein [25], membrane associated protein [26], porphyrin [19], components involved in the biosynthesis/metabolic pathways of fatty acid/lipids [27], ion channel [16], E3 ubiquitin ligase [28], a clathrin-associated adaptor protein [29], splicing factor 3b subunit 3 [30], putative MAPKKK [31], UDP-N-acetylglucosamine pyrophosphorylase 1 [31], AAA-type ATPase [32], Cullin domain protein [33], eukaryotic translation elongation factor 1 alpha (eEF1A)-like protein [34] and ferredoxin-dependent glutamate synthase [35]. These findings indicate that proteins regulating HR cell death are diverse. Despite these advances achieved, the molecular mechanism of cotton spontaneously inducing PCD is still elusive.

ATP-Citrate lyase (ATP-Citrate lyase, ACLY, also known as ACL) is mainly a cytoplasmic enzyme that exists in plants and animals with the hydrolysis of ATP [36]. In animals, ACL contains a polypeptide that is a homotetramer of 110–120 kDa subunits [37]. In plants, it is composed of two different subunits, ACLA and ACLB, and speculated that ACL is the form of A_4_B_4_ heterooctamer (4 A subunits and 4 B subunits base) [38]. Studies have shown that ACL plays a crucial role in human fetuses’ growth and development [39] and tumor cell proliferation in animals [40]. Studies in humans have also shown that inhibiting the activity of ACL would limit the aerobic glycolysis of cells, which in turn would arrest tumor growth [41]. Moreover, the activity of ACL is necessary for nutrient metabolism and histone acetylation [42]. The activity of ACL is also necessary for the normal growth and development in *Arabidopsis*. The reduced activity of *Arabidopsis* ACL would lead to a series of unhealthy phenotypes such as miniaturized organs, smaller cells, reduced cuticular wax deposition, aberrant plastid morphology [43]. A mutant of *OsACLA2* gene, *spl30–1*, was reported in rice and was identified as LMMs [44]. Hitherto, however, no phenotype caused by ACL gene mutation has been reported in cotton, and no ACL gene has been isolated in cotton.

Cotton (*Gossypium hirsutum*) is the most important natural fiber crop in the world and provides huge amounts of renewable fiber and oilseeds for all mankind. The cotton cultivar that accounts for more than 90% of production output is allotetraploid Upland cotton, which was formed in ~ 1–2 million years ago (Mya) through the hybridization and natural doubling between Old World A-genome progenitor and a New World D-genome ancestor [45, 46]. Therefore, the genetic information of the At subgenome and the Dt subgenome are contained in the allotetraploid cotton, and they complement each other in some traits. This phenomenon makes it difficult for genes controlling lesion mimics to be exposed in the cotton complex genome. As far as we know, the specific function of ACL in cotton has not been described. In this study, inhibiting the expression of ACLB by virus-induced gene silencing (VIGS) technology not only caused strong cell death in the newly-grown leaves and stem apexes of cotton but also activated the expression of salicylic acid (SA) signalling-related genes and PR genes. In *Arabidopsis*, T-DNA insertion mutation *AtACLB-2* alone enhanced the resistance to *V. dahliae* infection without causing strong cell death. It is worth noting that the expression level of ACLB in *Arabidopsis* is negatively correlated with the resistance of *V. dahliae* infection. We propose that ACLB plays a negative regulatory role in inducing cell death and innate immunity of cotton, which provides theoretical guidance for developing cotton varieties with resistance against Verticillium wilt (VW).

## Results

### ACL genes with high amino acid identity in cotton

**Fig. 1:**
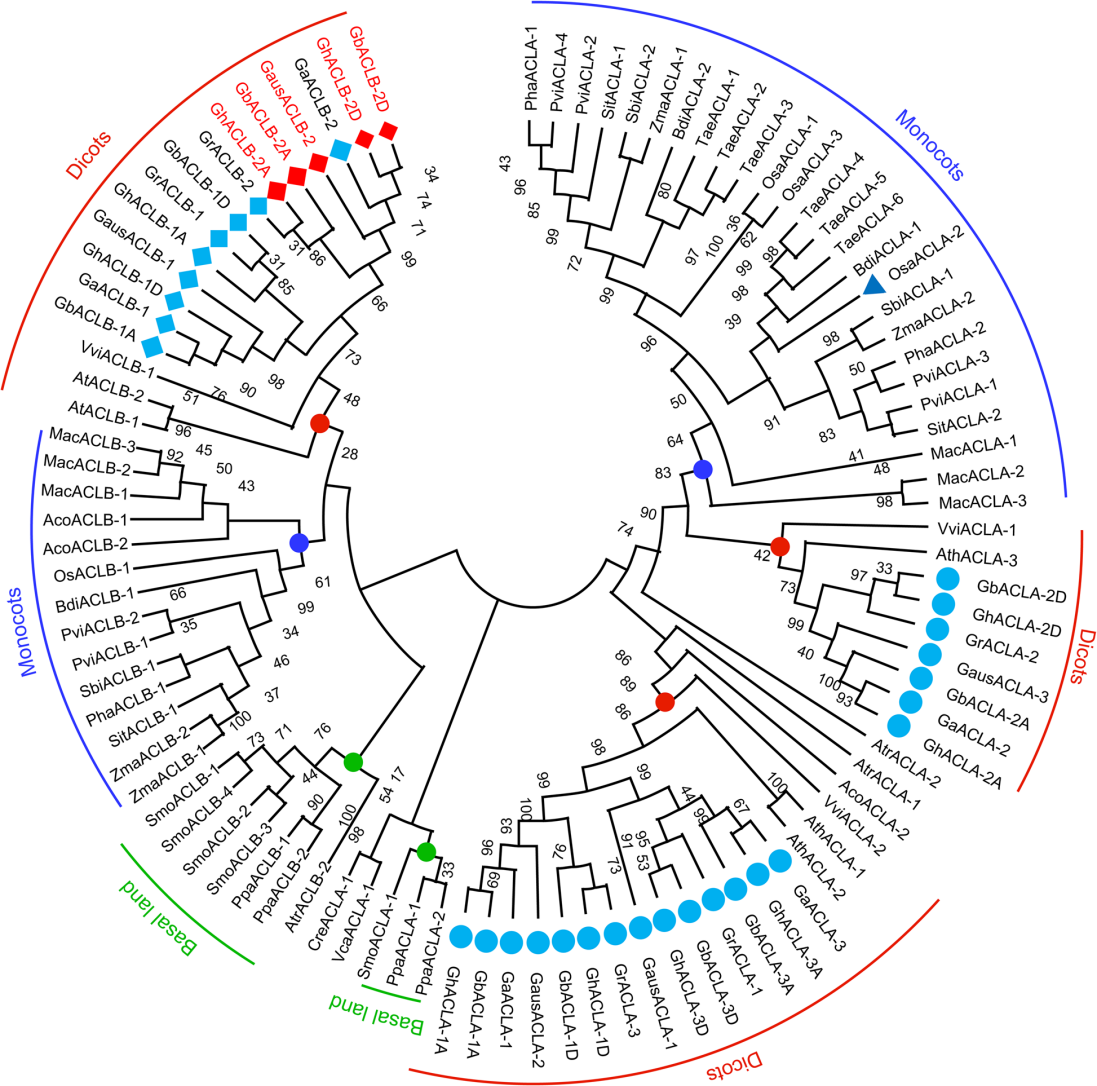
Phylogenetic analysis revealed the protein sequence divergence of ATP-citrate lyase (ACL) genes in major plant species. The full-length of ACLA and ACLB protein sequences of 22 plants were aligned to construct a maximum likelihood tree. 1000 bootstrap re-samplings were used in each node. The dicots, monocots and basal land clades are respectively colored in red, blue and green dots. The ACL genes of *Gossypium* are indicated by cyan and red nodes, where ACLA and ACLB are indicated by circles and diamonds, respectively. Species abbreviations are as follows. Aco: *Ananas comosus*, Ath: *Arabidopsis thaliana*, Atr: *Amborella trichopoda*, Bdi: *Brachypodium distachyon*, Cre: *Chlamydomonas reinhardtii*, Gaus: *Gossypium australe*, Ga: *Gossypium arboreum*, Gb: *Gossypium barbadense*, Gh: *Gossypium hirsutum*, Gr: *Gossypium raimondii*, Mac: *Musa acuminata*, Osa: *Oryza sativa*, Pha: *Panicum hallii*, Ppa: *Physcomitrella patens*, Pvi: *Panicum virgatum*, Sbi: *Sorghum bicolor*, Sit: *Setaria italica*, Smo: *Selaginella moellendorffii*, Tae: *Triticum aestivum*, Vca: *Volvox carteri*, Vvi: *Vitis vinifera*, Zma: *Zea mays*

In plants, ACL consists of two different subunits, subunit A and subunit B [36]. There are three and two loci discovered in the *Arabidopsis* genome encoding ACLA and ACLB subunits respectively [36]. ACLA protein is produced by the differentiation of SCS β-subunit while ACLB is evolved through the gene fusion form combined α-subunit of succinyl-CoA synthase (SCSα) and citrate synthase (CS) [36]. To identify the ACL homolog in *Gossypium*, the full-length amino acid sequence of AtALCA and AtACLB was used as the query in Pfam (Version 31, http://pfam.xfam.org) to search the seed sequences of conserved domains. The search results indicate that ACLA contains ATP-grasp 2 and citrate binding domains, while ACLB contains CoA_ligase and Citrate_synthase domains. Then, the identified conserved domains were used as queries to search for further ACL proteins in the *G. australe*, *G. arboreum*, *G. barbadense*, *G. hirsutum* and *G. raimondii* genome. The presence of ACLA and ACLB conserved domains in each hit protein sequence was further verified by hmmscan (http://hmmer.org) against the Pfam-A profile HMM database (e-value cutoff = 1). In this way, 21 ACLA homologous sequences and 14 ACLB homologous were totally identified in two tetraploid (AADD genome) and three diploid (A, D or G genome, respectively) *Gossypium* species (Data S1). And ACLs were named according to the position distribution of homologs in each genome (Table S1). To better understand the evolutionary relationships between different members of the *Gossypium* ACL gene family, ACLA and ACLB genes were identified in 17 plant genomes, including ten monocots, three dicots, two basal land plants and two algae. In total, 60 ACLA homologous sequences and 38 ACLB homologous sequences were subjected to a maximum likelihood phylogenetic analysis (Fig. 1).

The resulting phylogenetic tree shows that the ACLA genes of monocot and dicot plants are descendants of their ancestor gene homologs. There are three ACLA homologues in *Gossypium*, which are divided into two independent clades isolated from monocots, indicating possible differences in their functions. The ACLB gene was classified into four large clades, and the ACLB gene have the same ancestral gene in *Gramineae* (*Poaceae*). There are two ACLB homologues in *Gossypium*. The cotton ACLB gene and the *Arabidopsis* ACLB gene share a common ancestral homologous gene, and have been separated from basic land plants and monocotyledonous plants. In addition, no homologues of ACLB genes were found in *Triticum aestivum*, but six homologues of ACLA genes were identified, suggesting the functional diversity of ACLA genes in wheat.

### Sequence analysis of the ACLB-2 gene in *Gossypium*

The most widely-cultivated cotton (*Gossypium hirsutum* L.) is an allotetraploid species (AADD genome), being derived from interspecific hybridization between its two closest diploid ancestor species, *G. arboreum* (A genome) and *G. raimondii* (D genome), in 1–2 Mya [47, 48]. In this study, three ACLA genes and two ACLB genes were identified in both Upland cotton (AD_1_) and Sea Island cotton (AD_2_). The ACLB genes of *G. australe, G. hirsutum* and *G. barbadense* were cloned from the respective cDNA library. Sequence analyses revealed that *GausACLB-2* spans 4056 bp with 15 exons and the encoded polypeptide is composed of 608 amino acid residues with molecular mass of 65 kDa (Fig. S1a). In *Arabidopsis*, the ACLA subunit is encoded by three genes (AT1G10670, AT1G60810 and AT1G09430), and the ACLB subunit is encoded by two genes (AT3G06650 and AT5G49460) [36]. Comparative analysis found that *GausACLB-2* and *GausACLB-1* have the same number of exons as *AtACLB-2,* but with one exon less than *AtACLB-1*. The 12th exon of *GausACLB* was split into two exons. In addition, the second intron of *GausACLB* is eight times longer than that of *AtACLB* (Fig. S1a).

**Fig. 2:**
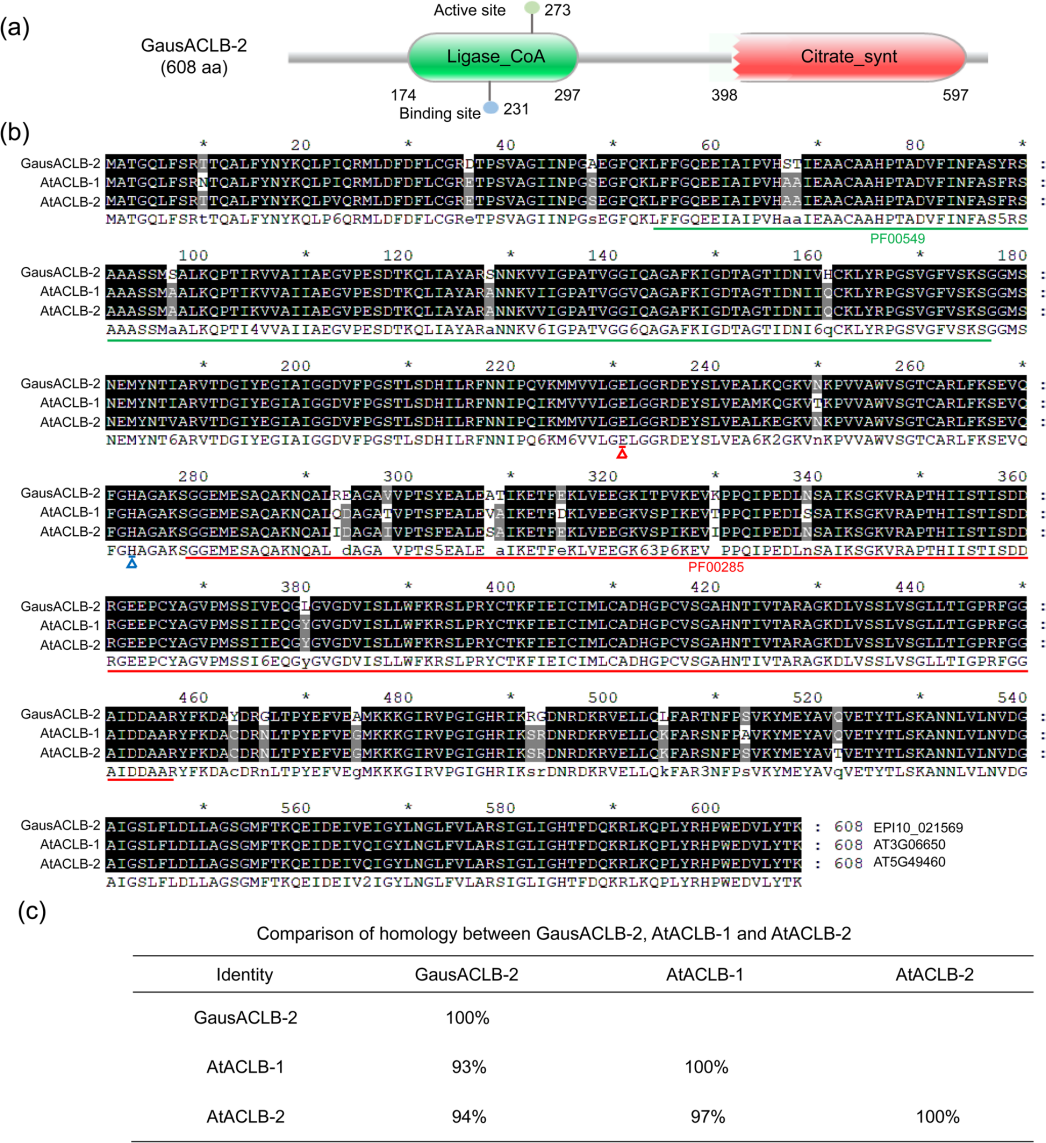
Primary structure analysis of GausACLB-2. **a** The conserved domain of GausACLB-2 protein. The N-terminal CoA_ligase (PF00549) domain and the C-terminal Citrate_synthase (PF00285) domain. Conserved domains are predicted using SAMRT and pfam. The binding site and activation site on the CoA_ligase domain are determined by uniprot database. **b** Protein sequence alignment of *GausACLB-2*, *AtACLB-2* and *AtACLB-2* genes. The red and blue *triangles* below the residues show the *Magnesium metal binding* site and *Tele-phosphohistidine intermediate active* site, respectively. PF00549, CoA_ligase; PF00285, Citrate_synthase. **c** The protein homology among GausACLB-2, AtACLB-1 and AtACLB-2

Amino acid sequence analysis showed that cotton ACLB proteins contain the conserved domains of CoA_ligase and Citrate_synthase except *GbACLB-1A* (Fig. S1b). There are Magnesium metal binding site and Tele-phosphohistidine intermediate active site on the Ligase_CoA domain (Fig. 2a). Compared with the two ACLB amino acid sequences of *Arabidopsis thaliana*, *GausACLB-2* has the highest identity with 93% of *AtACLB-1* and 94% of *AtACLB-2*, respectively (Fig. 2b, c).

### ACLB-2 is a constitutively expressed on plasma membrane in cotton

To characterize the expression characteristics of *GausACLB-2* gene, a sequence of 2000 bp upstream of the gene start codon was cloned, and the Neural Network Promoter Prediction [49] was used to predict the transcription start site (TSS). Four TSSs were predicted to be A at − 1187, A at − 1166, A at − 731, and C at − 489, with the scores of 0.97, 0.94, 0.81, and 0.99, respectively (Fig. S2a). PlantCARE [50] software was used to predict the potential cis-regulatory elements in the promoter region, define the A base of the gene start codon as + 1, and construct a map of promoter cis-regulatory elements (Fig. S2b). The promoter region includes the expression control elements TATA-Box and CAAT-Box, which are necessary for basic promoters of higher plants, at positions − 518/− 513 and − 551/− 548, respectively. In addition to the core regulatory elements, the promoter of *GausACLB-2* is also rich in cis-regulatory elements. Among them, TCA-element at − 1898/− 1890; ABRE cis-regulatory element related to hormone regulation at − 600/− 596; ACE at − 1961/− 1953; Box 4 at − 1285/− 1280; chs-Unit 1 m1 at − 1104/− 1095, and a CAT-box at − 419/− 414 with regulatory elements related to plant growth regulation and light response (Table S2). The analysis of promoter regulatory elements suggests that the expression of *GausACLB-2* is regulated by diverse biological processes.

**Fig. 3:**
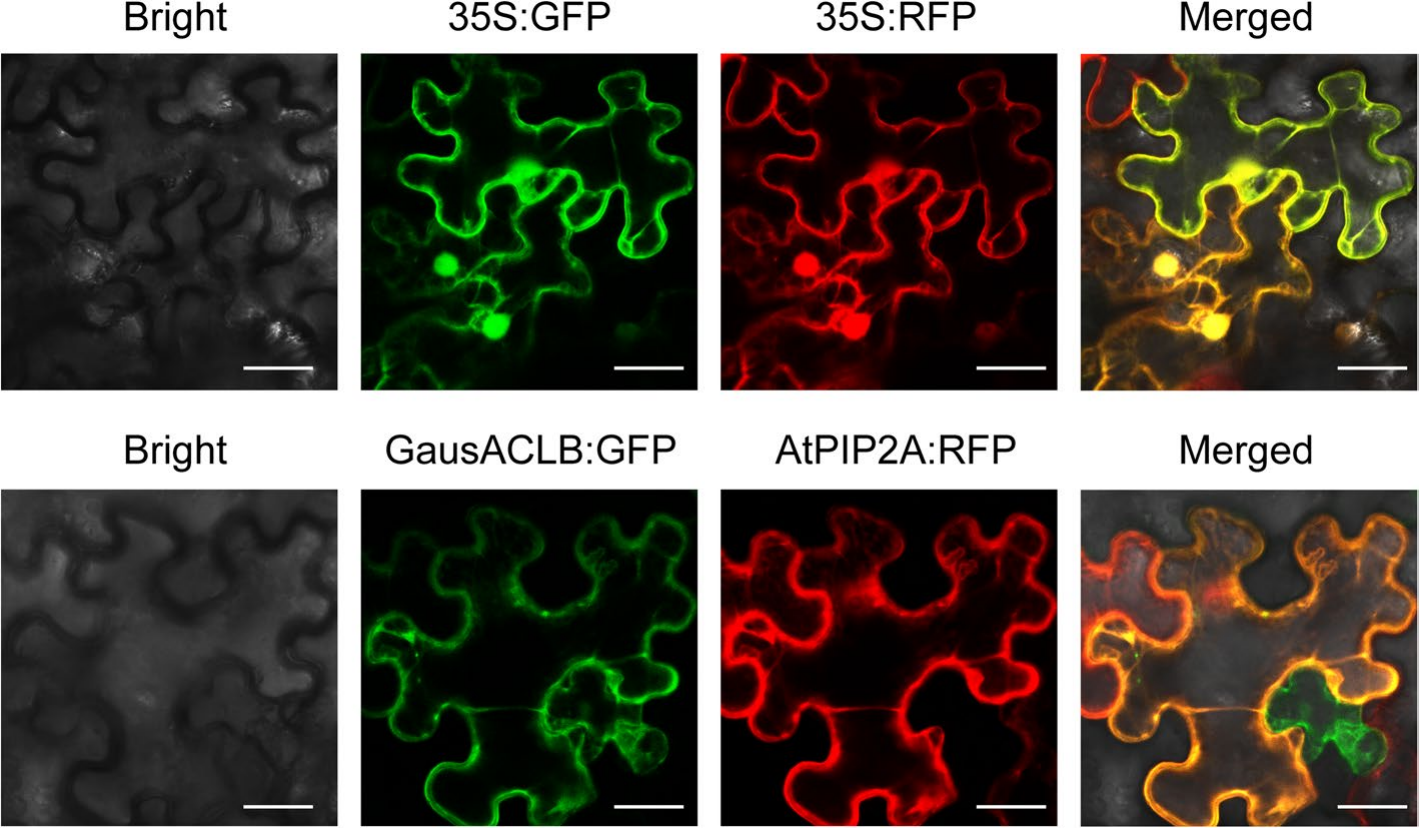
Subcellular localization of GausACLB-2 protein in tobacco epidermal cells. Fluorescent signals from GausACLB-GFP and plasma membrane marker AtPIP2A-RFP expressed in tobacco leaf epidermal cells. Marker proteins were co-transformed with GausACLB-GFP. Transient expression and microscopy observation for protein localization signals were repeated for three times. Bar, 50 μm

GausACLB-2 and GFP fusion protein was first cloned and driven by the cauliflower mosaic virus 35S promoter. GausACLB-2 and GFP fusion protein was transiently expressed in *Nicotiana benthamiana* epidermal cells to determine the subcellular localization of GausACLB-2 protein. Co-expression with the plasma membrane marker AtPIP2A-RFP revealed that GausACLB-2-GFP proteins co-localized with the marker (Fig. 3). These data suggest that the GausACLB-2 proteins are located on plasma membranes.

**Fig. 4:**
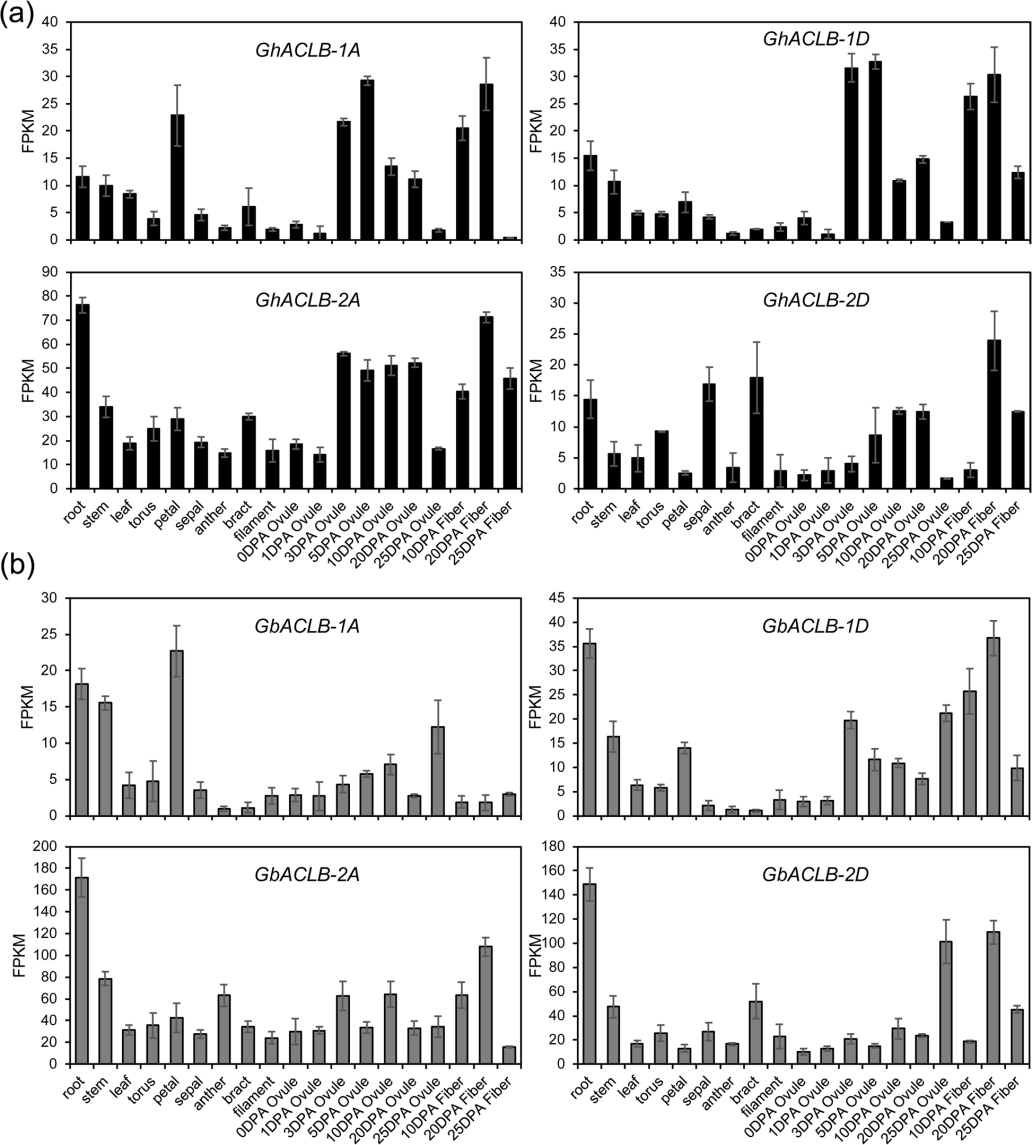
ACLB expression showed constitutive in 11 tissues of cotton. **a** TM-1 (*G. hirsutum*). **b** Hai7124 (*G. barbadense*). Data are shown as mean ± SD

To clarify the expression patterns of ACLB genes in cotton, the RNA-seq data of 19 tissues in Upland cotton “TM-1” and Sea Island cotton “Hai7124” were investigated. *GhACLB-2A* was a constitutively highly expressed gene in all 19 analyzed tissues of TM-1. Moreover, *GhACLB* genes had a high expression level in root, stem and leaf (Fig. 4a). *GbACLB* genes of Hai7124 had a similar expression pattern to *GhACLB*, a slight difference was *GbACLB* were predominantly expressed in root (Fig. 4b). In general, both *GhACLB* and *GbACLB* were constitutively expressed genes in cotton. The results of quantitative reverse transcription PCR (qRT-PCR) also showed that *GausACLB-2*, *GhACLB-2* and *GbACLB-2* were expressed in root and leaf (Fig. S3).

### Silencing ACLB led to cell death at newly-grown leaves and stem apexes in cotton plants

To determine whether ACLB genes were functionally differentiated in cotton, the tobacco rattle virus (TRV)-based virus-induced gene silencing (VIGS) strategy was used to knock down the transcripts of *GhACLB* and *GbACLB*. Due to the high homology of the two cotton gene sequences encoding ACLB subunits (Table S3), the ACLB-1 or ACLB-2 genes cannot be silenced alone. In addition, considering the high sequence similarity between ACLB paralogs of the At- and the Dt- subgenome in tetraploid cotton, the pair of homologous genes were also considered as a single unit in this research. For each group, the conserved Citrate_synthase domain was used in VIGS. The constructed pTRV2 vectors that consisted of conserved coding sequences of *GhACLB* members was named as TRV: *GhACLB* and the pTRV2 vector containing the conserved coding fragments of *GbACLB* members was named TRV: *GbACLB*. The pTRV2 vector without sequence insertion (TRV: 00) was used as a negative control and pTRV2 with a fragment of *GhCLA1* (TRV: *CLA*) was as VIGS efficiency indicator. The 10-day-old cotton seedlings were treated according to standard VIGS experimental procedures [51].

**Fig. 5:**
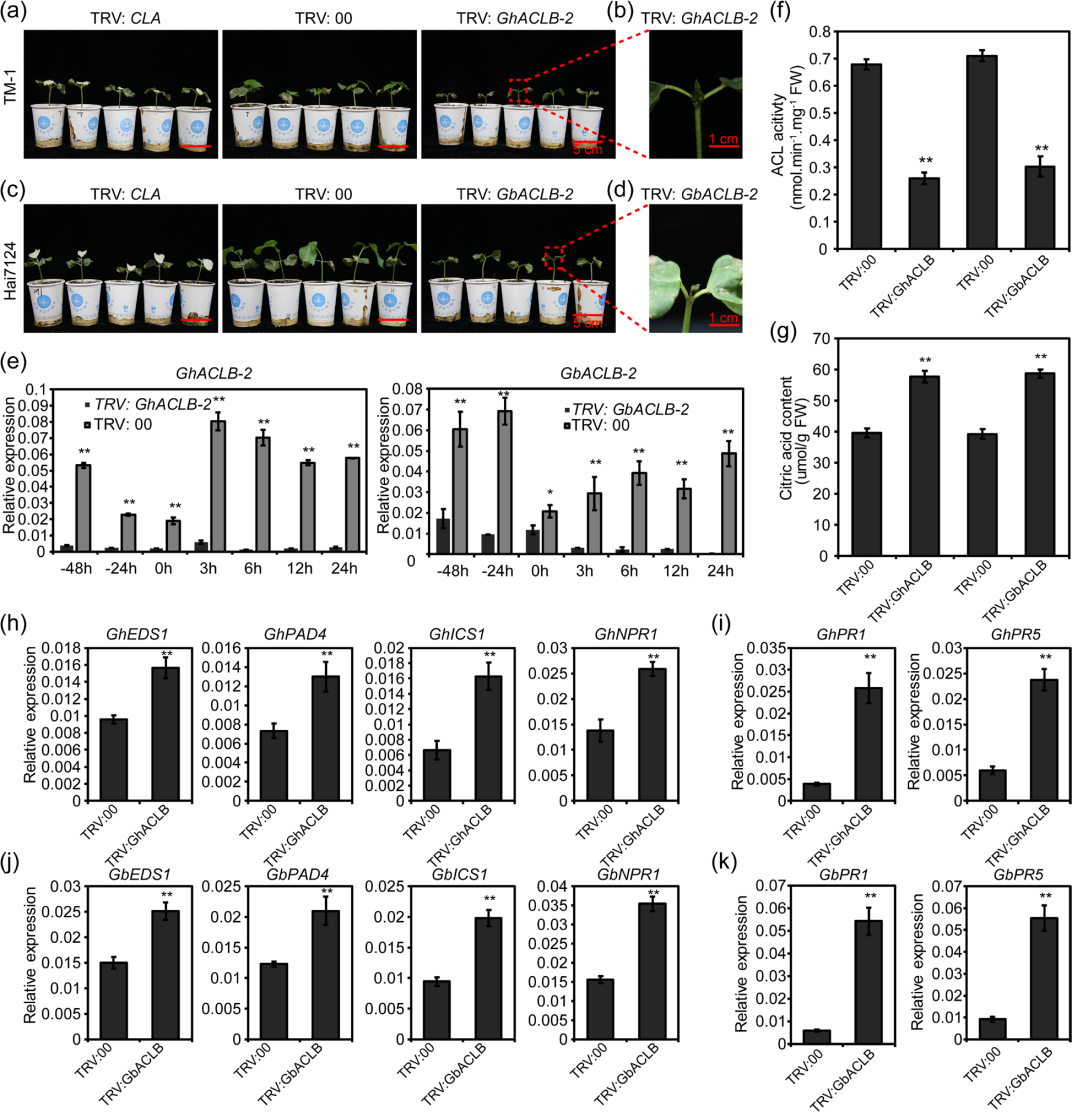
Silencing ACLB gene spontaneously induced strong cell death phenotypes in cotton. **a** TM-1 (*G. hirsutum*) plants. Ten-days-old cotton plants were infiltrated with *Agrobacterium* cells carrying the VIGS vector. Silencing the *GhACLB* gene induced programmed cell death in the new leaves and stem apexes of cotton. Photographs were taken at 2 weeks after infiltration. The experiments were repeated three times and similar results were obtained. Bar, 5 cm. **b** Enlarged images of the section outlined by red dashed lines in (**a**). Bar, 1 cm. **c** Hai7124 (*G. barbadense*) plants. Silencing the *GbACLB* gene also induced cell death in the new leaves and stem apexes of cotton. The experiments were independently repeated three times and similar results were obtained. Bar, 5 cm. **d** Enlarged images of the section outlined by red dashed lines in (**b**). **e** When TRV: *CLA* plants showed albino phenotype, the time on the 14th day of VIGS treatment was appointed as 0 h, the relative expression levels of ACLB gene in the control were higher than in VIGS plants. **f** The leaf ACL enzyme activities in TRV:00 were higher than in TRV: *GhACLB* or TRV: *GbACLB* plants after 2 weeks of VIGS. FW, fresh weight. **g** Leaf contents of citric acid in TRV:00 were lower than in TRV: *GhACLB/*TRV: *GbACLB* plants after 2 weeks of VIGS. FW, fresh weight. **h** The relative gene expression levels of SA signal transduction pathway were lower in TRV:00 than in TRV: *GhACLB* plants. **i** The relative expression patterns of PR genes were lower in TRV:00 than in TRV: *GhACLB* plants. **j** The relative gene expression levels of SA signal transduction pathway were lower in TRV:00 than in TRV: *GbACLB* plants. **k** The relative expression levels of PR genes were lower in TRV:00 than in TRV: *GbACLB* plants. Data are shown as mean ± SE of three biological replicates; asterisks indicate statistically significant differences, as determined by the Student’s *t*-test (*P < 0.05, **P < 0.01)

After 2 weeks of treatment, TRV: *CLA* plants showed the expected albino phenotype. Simultaneously, the wilting appeared from the newly-grown leaves and stem apexes of TRV: *GhACLB* and TRV: *GbACLB* plants (Fig. 5a-d). The phenomena indicated that silencing ACLB genes could induce cell death in both TM-1 and Hai7124. Further analyses were performed for the ACLB gene expression levels in TM-1 and Hai7124 by qRT-PCR (Fig. 5e). Compared with TRV: 00 plants, the plants containing TRV: *GhACLB* or TRV: *GbACLB* had significantly reduced ACLB gene expression at seven time-points.

To determine whether ACL activity was attenuated in ACLB-silenced plants, the enzyme activity assays were performed. As expected, the total ACL activities were significantly reduced in TRV: *GhACLB* and TRV: *GbACLB* plants compared to the TRV: 00 plants, respectively (Fig. 5f). Since citric acid is a direct substrate of ACL, the citric acid content was subsequently investigated in ACLB-silenced plants. The contents of citric acid in TRV: *GhACLB* and TRV: *GbACLB* plants were significantly higher than that in TRV: 00 plants (Fig. 5g), indicating that the ACL activities were attenuated by suppressing the expression of ACLB in cotton. Given that SA signaling pathway plays an essential role in the HR [8], the transcript levels of SA signalling-related genes were monitored. The expression levels of EDS1, PAD4, NPR1 and ICS1 were significantly different between the TRV: ACLB and TRV: 00 plants (Fig. 5h, j), indicating the genes related to SA pathway were activated in ACLB-silenced plants. Furthermore, the transcript levels of PR genes (*PR1* and *PR5*) were higher in ACLB-silenced plants than in TRV: 00 plants (Fig. 5i, k). These data suggest that knockdown of ACLB led to activation of HR-like cell death in cotton seedlings.

### Knockdown of ACLB induced the expression of senescence-related genes and the accumulation of reactive oxygen species

**Fig. 6:**
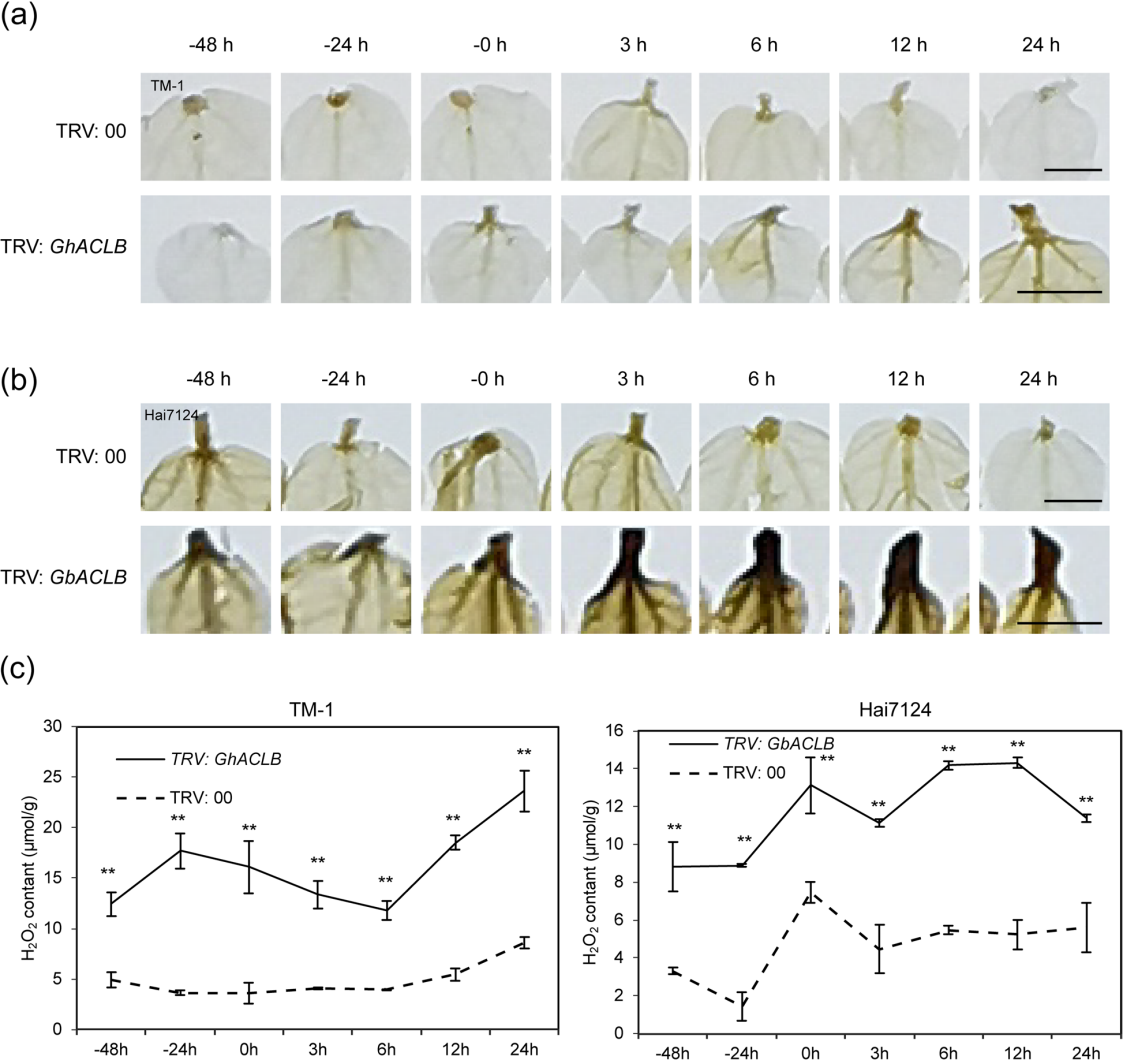
Knockdown of ACLB caused the significant accumulation of reactive oxygen species at the cell death site. **a** H_2_O_2_ accumulation at the lesion mimic site of the control and VIGS-TM-1 plants was visualized through 3,3′-diaminobenzidine (DAB) staining. Scale bars, 0.5 cm. **b** H_2_O_2_ accumulation at the lesion mimic site of control and VIGS-Hai7124 plants was visualized through DAB staining. Scale bars, 0.5 cm. **c** Measurements of the H_2_O_2_ contents in the lesion mimic cotton leaves. Data are shown as mean ± SE of three biological replicates (**P < 0.01, Student’s *t*-test)

Over accumulation of hydrogen peroxide (H_2_O_2_) in plant cell is regarded as a characteristic of a typical cell death response [44]. To evaluate whether the cell death phenotype in ACLB-silenced plants was associated with H_2_O_2_ accumulation, the H_2_O_2_ level were determined. In this study, the newly-grown leaves of the plants were used to determine the accumulation of reactive oxygen species (ROS) at the aforementioned seven time-points during VIGS treatment. By incubating newly-grown leaves from plants containing TRV: 00 (CK), TRV: *GhACLB* and TRV: *GbACLB* in 3, 3′-diaminobenzidine (DAB) for 8 h, brown coloration was developed on leaves from ACLB-silenced plants containing TRV: *GhACLB* or TRV: *GbACLB* but not from CK (Fig. 6a, b). Further quantitative analysis showed that the ACLB-silenced plants produced significantly much more H_2_O_2_ than CK. A higher concentration of H_2_O_2_ appeared in Hai7124 from 0 h (albino leaf appeared in the positive control plants) to 12 h while the gradual increment of H_2_O_2_ generated in TM-1 after 6 h (Fig. 6c).

*SGR*, *WRKY23* and *Osl57* are recognized as transcription factors related to senescence, which are specifically expressed during the PCD process of plants [44]. The expression of the above three transcription factors, *SGR*, *WRKY23* and *Osl57*, were analyzed using qRT-PCR. The results showed that they expressed significantly higher in ACLB-silenced plants than that in TRV: 00 plants (Fig. S4a, b), which indicated that silencing the ACLB genes induced the expression of *SGR*, *WRKY23* and *Osl57* in TM-1 and Hai7124 plants.

Given that *OsACLA-2* negatively regulating innate immune responses in rice [44] and given knockdown of ACLB led to activation of HR-like cell death in cotton seedlings, which prompted us to examine whether ACLB had a function in disease resistance. However, ACLB-silenced plants induced strong cell death in newly-grown leaves and stem apexes, which led to the failure of subsequent fungal pathogenicity assays. To confirm the role of ACLB gene in participating in plant immune response, the dicotyledonous model plant *Arabidopsis* was selected for functional verification of ACLB gene.

### *GausACLB-2* negatively affected disease resistance to *V. dahliae*

To determine whether the ACLB-2 gene is involved in the resistance to *V. dahliae*, *Arabidopsis* T-DNA insertion mutant *aclb-2* (SALK_138734) was used for gene function validation of resistance to *V. dahliae*. The triple primers, namely, two forward primers and one sharing reward primer, were used for PCR amplification to detect the T-DNA insertion events of *aclb-2* mutants (Fig. S5a, b). The results showed that the T-DNA insertion in the promoter region made *AtACLB-2* express significantly lower than wild-type (WT) (Fig. S5c), and also caused the root length of 14-day-old seedlings to be significantly shorter than that of WT (Fig. S5d, e).

**Fig. 7:**
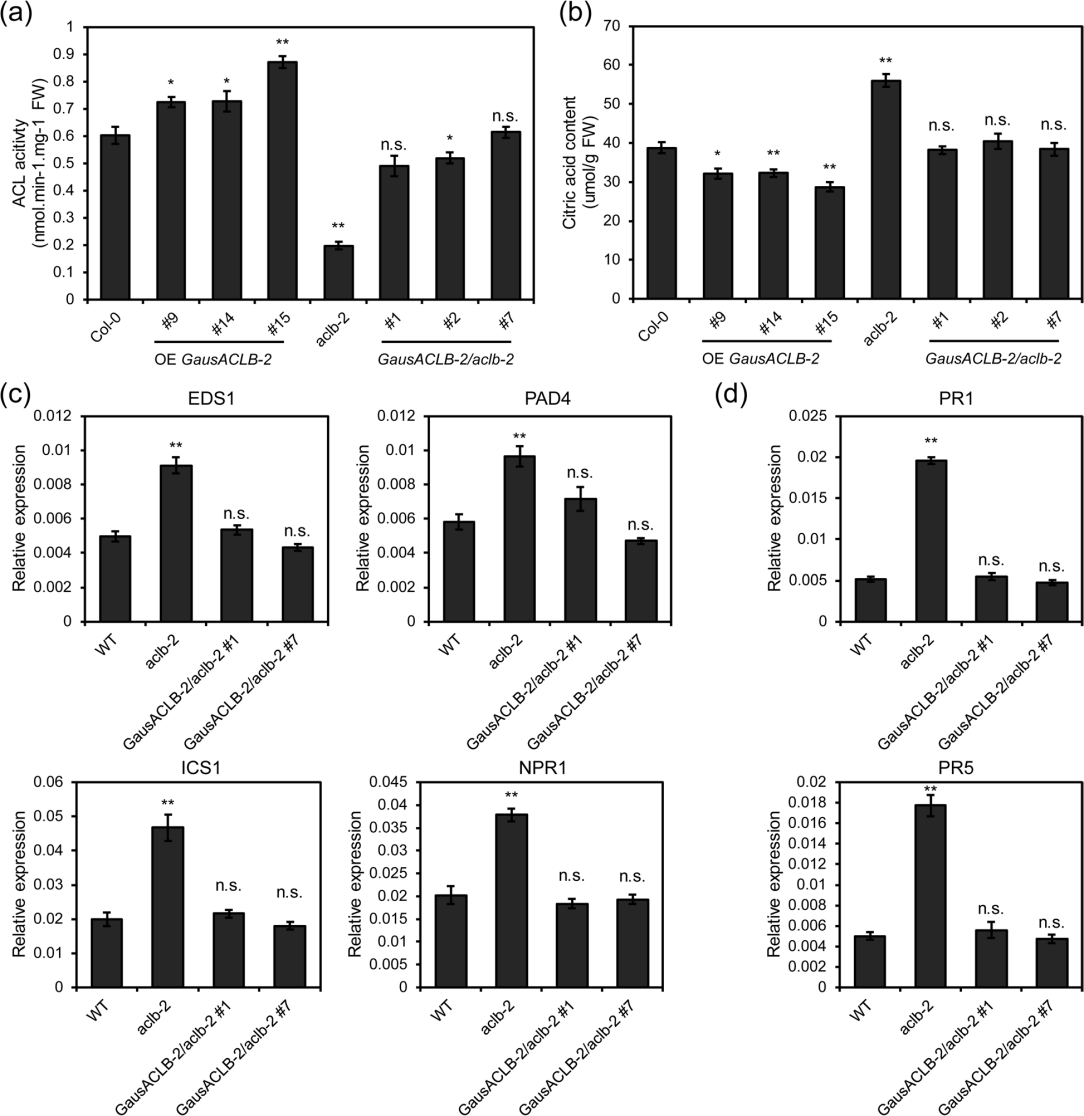
Reduced ACL activities in *aclb-2* mutants activates SA-mediated defense response. **a** ACL activity in leaves of Col-0, OE-*GausACLB-2*, *aclb-2* and complementation lines at four-week-old stage. FW, fresh weight. **b** Leaf contents of citric acid in Col-0, OE-*GausACLB-2*, *aclb-2* and complementation lines at four-week-old stage. FW, fresh weight. **c** SA signal transduction pathway genes expression level in WT, *aclb-2* and the indicated transgene *Arabidopsis*. WT, wild type (Col-0). **d** Expression patterns of PR genes in WT, *aclb-2* and the indicated transgene *Arabidopsis*. WT, wild type (Col-0). Data are shown as mean ± SE of three biological replicates; asterisks indicate statistically significant differences, as determined by the Student’s *t*-test (*P < 0.05, **P < 0.01, n.s., not significant)

To better understand the function of ACLB-2 gene in VW resistance, *GausACLB-2* was transferred to *Arabidopsis* with Col-0 or *aclb-2* as backgrounds to produce overexpressing (OE) and function-restoring *Arabidopsis*. To this end, we first investigate whether ACL activity was affected in *aclb-2* mutants. The results indicated that the ACL activities were significantly reduced in *aclb-2* mutants compared to wild-type (WT) plants (Fig. 7a). Three independent OE-GausACLB-2 transgenic lines (#9, #14 and #15) exhibited enhanced ACL activity compared with WT plants, while the ACL activity was comparable between complementation lines (#1, #2 and #7) and WT plants (Fig. 7a). Moreover, the contents of citric acid in *aclb-2* mutants were significantly higher than that in WT (Fig. 7b).

**Fig. 8:**
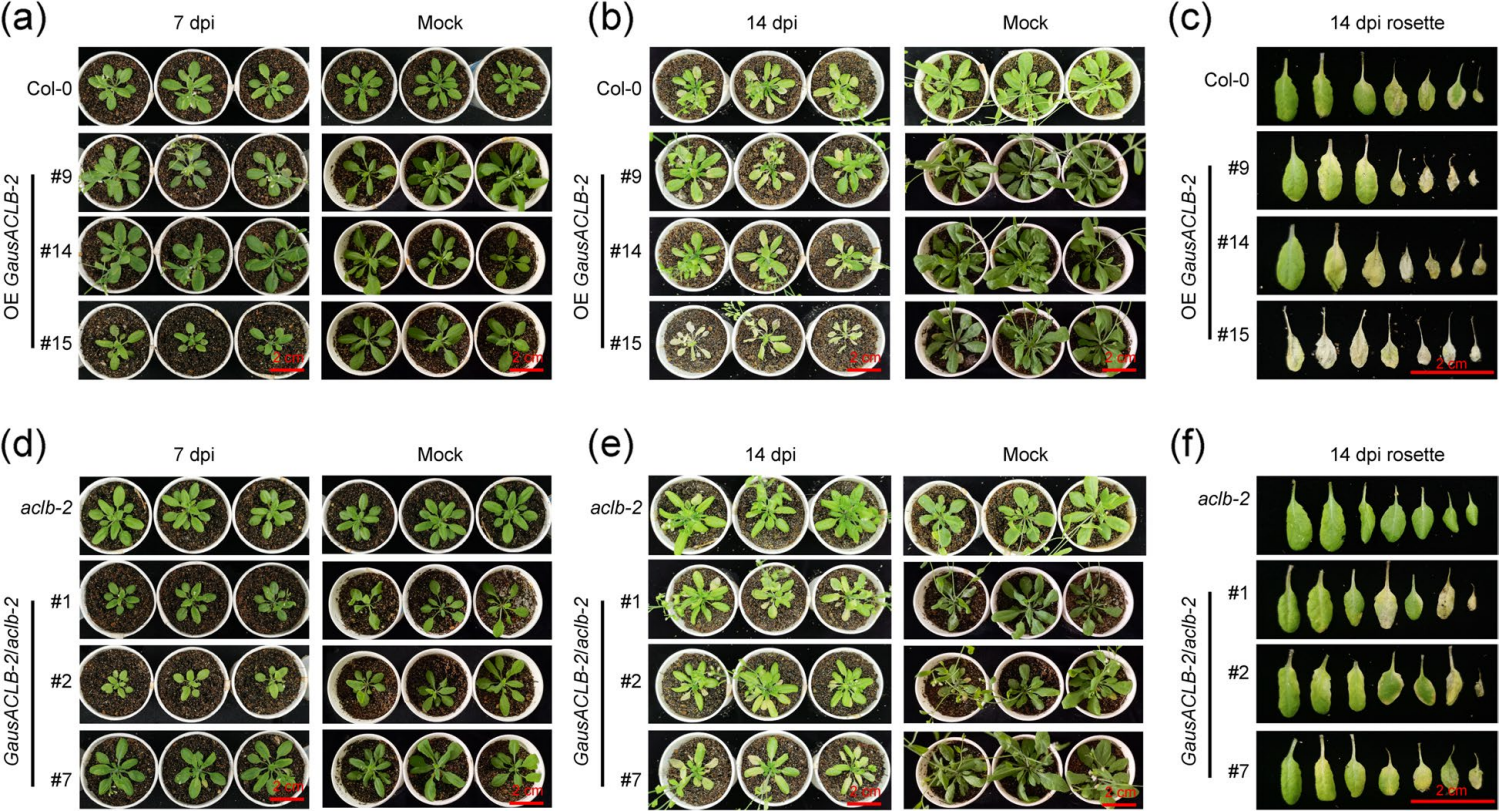
*GausACLB-2* negatively regulated *Arabidopsis* resistance to *Verticillium dahliae*. **a** Disease symptoms of WT and indicated OE-*GausACLB-2 Arabidopsis* inoculation with *V. dahliae* strain V991 at 7 dpi. Sterile water treatment was used as mock. Bar, 2 cm. **b** Disease symptoms of WT and indicated OE-*GausACLB-2 Arabidopsis* inoculation with *V. dahliae* strain V991 at 14 dpi. Sterile water treatment was used as mock. Bar, 2 cm. **c** Disease symptoms of rosette leaves in indicated *Arabidopsis* plants inoculation with V991 at 14 dpi. Bar, 2 cm. **d** Disease symptoms of complementation lines and mutants *Arabidopsis* (*aclb-2*) inoculation with *V. dahliae* strain V991 at 7 dpi. Sterile water treatment was used as mock. Bar, 2 cm. **e** Disease symptoms of complementation lines and mutants *Arabidopsis* (*aclb-2*) inoculation with *V. dahliae* strain V991 at 14 dpi. Sterile water treatment was used as mock. Bar, 2 cm. **f** Disease symptoms of rosette leaves in indicated *Arabidopsis* plants inoculation with V991 at 14 dpi. Bar, 2 cm. The *V. dahliae* infection assays was repeated independently three times with similar results

Consistent with the ACL activity assay results, the expression levels of SA signalling-related genes, such as EDS1, PAD4, NPR1 and ICS1, were found to be significantly enhanced in *aclb-2* mutant plants compared with WT plants while they had no significantly difference between complementation lines and WT plants (Fig. 7c). Furthermore, the transcript levels of PR genes (*PR1* and *PR5*) were higher in *aclb-2* mutant plants than in WT plants (Fig. 7d), and the transcript levels of *PR1* and *PR5* in complementation lines were indistinguishable from WT plants. These data suggest that suppressing ACLB-2 activates SA-mediated defense response. Subsequently, the VW resistance was evaluated. The results showed that overexpression of *GausACLB-2* made *Arabidopsis* plants be more sensitive to *V. dahliae* than their recipients either in Col-0 or in *aclb-2* (Fig. 8). In contrast, *aclb-2* showed stronger resistance to VW compared with Col-0.

**Fig. 9:**
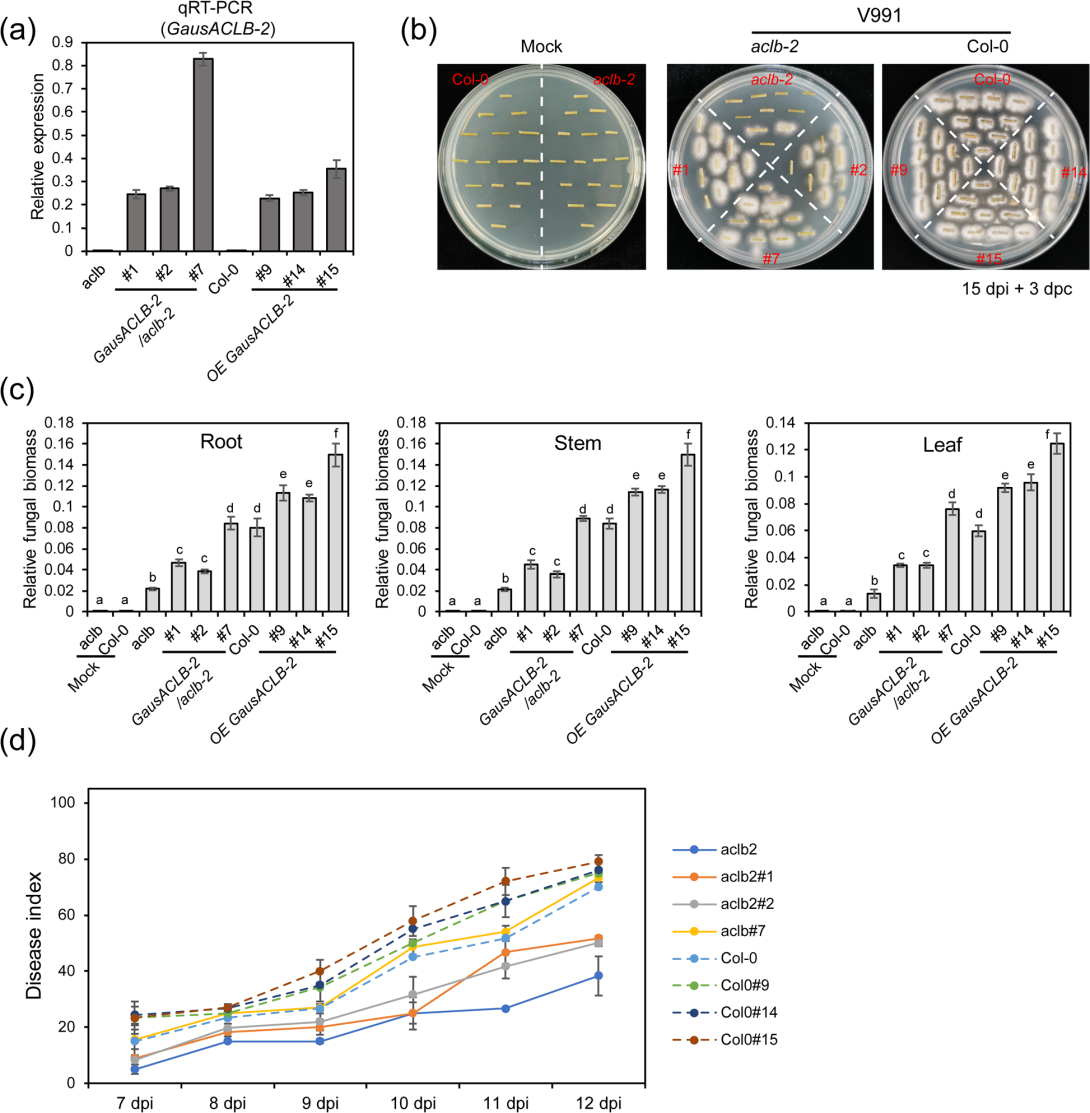
The expression level of *GausACLB-2* in *Arabidopsis* is negatively correlated to *V. dahliae* resistance. **a** Relative expression levels of *GausACLB-2* in the overexpression and complementation lines. **b** Fungal recovery assays in overexpression and complementation lines 3 days after recovery. **c** Quantification of fungal biomass. After 15 days of inoculation, DNA was extracted from plant root, stem and leaf, and the biomass of *V. dahliae* was analyzed using specific primers. The marked letter method was used to display the results of statistical analysis (**P < 0.01, Student’s *t*-test). **d** Disease index of transgenic *Arabidopsis*. Data are shown as mean ± SE of three biological replicates

The fungal recovery and relative fungal biomass assays indicated that much more fungal biomass appeared in OE9, OE14 and OE15 plants compared to Col-0 plants (Fig. 9a, b, c). The disease index also confirmed that overexpression of *GausACLB-2* weakened the resistance to *V. dahliae* in *Arabidopsis* (Fig. 9d). Compared with *aclb-2*, more fungal biomass n was also found in the three function-restoring plants, and in their different tissues (Fig. 9b, c). In addition, the function-restoring plants also showed a higher disease index compared with *aclb-2* (Fig. 9d).

By comparing the resistance performance of *aclb-2*, col-0, OE and function-recovering plants, it was validated that ACLB-2 negatively affected the resistance to *V. dahliae*. For example, when *ACLB-2* gene was transformed in the *aclb-2* background (OE ACLB-2/aclb-2), its disease resistance gradually decreased with the expression level of *GausACLB-2* increasing (Fig. 9). When *ACLB-2* gene was transformed in the Col-0 (wild-type of *Arabidopsis*) background (OE ACLB-2), the disease resistance also gradually decreased (Fig. 9). Notably, due to the endogenous *AtACLB-2* gene, the overall expression level of ACLB-2 in Col-0 background was higher than OE ACLB-2/aclb-2, leading to that the plants containing both *GausACLB-2* and *AtACLB-2* (OE ACLB-2) were much more sensitive to *V. dahlia* than the plants having *GausACLB-2* alone (OE ACLB-2/aclb-2) (Fig. 9). Thus, these results suggested that *GausACLB-2* negatively affected resistance to *V. dahliae*.

## Discussion

Acetyl-CoA (CoA) is a central metabolic intermediate and determines the balance between cellular catabolism and anabolism [52]. In plant cells, various biomolecule synthesis supported by CoA is important for growth, development and protection of plants [53]. In vertebrates and insects, it shows that acetyl-CoA precursor is produced by ATP-citrate lyase (ACL) [36], and ACL has been well studied in animals [39]. According to our ACL phylogenetic analysis, some plants lack ACLA or ACLB, indicating that there may be a variety of acetyl-CoA metabolic pathways in plants. In this study, it was also revealed that the ACLA subunit of cotton is encoded by three genes, while the ACLB subunit is encoded by two genes (Fig. 1). In addition, we discovered new functions of ACLB in cell death and immune response.

ATP citrate lyase converts citrate to acetyl-CoA, where it regulated activity of histone acetyl transferases (HATs) by controlling the availability of acetyl-CoA [42]. Depletion of ACL from cultured human colon carcinoma cells specifically decreased histone acetylation in the nucleus [42]. Also, loss of ACL in cultured mouse cells reduced the increase in histone acetylation and inhibited the increase in expression of specific genes [42]. Thus, ACL activity is required to link metabolic activity and gene expression. Here, we found that impaired expression of ACLB resulted in ACL activities were significantly reduced and activation of the HR-like cell death phenotype in cotton seedlings (Fig. 5). Furthermore, the SA singling pathway and PR genes were activated in ACLB-silenced cotton and *aclb-2* mutants (Figs. 5 and 7). Thus, it is possible that reduced ACL activity cause serious metabolic disorders that alter histone acetylation and gene expression, ultimately leading to a cell death phenotype.

PCD is an important form to protect plants from biotic or abiotic stress [54]. Plant HR is a rapid localized cell death triggered by activation of immune receptors upon pathogen recognition [8]. The NLRs detect pathogen effectors either directly or indirectly and activate a robust immune response that includes HR cell death in plant [55]. For example, ZAR1 resistosome can switch conformation to expose a funnel-shaped structure that acts as a calcium-permeable cation channel to trigger HR cell death [12, 13]. Although PCD plays an important role in various developmental and physiological functions, the current understanding of the mechanism of PCD in plants is still limited [56–58]. Lesion mimic mutants (LMMs) spontaneously produce necrotic lesions and activate defense responses, being considered to be an effective tool for understanding the cellular mechanisms governing PCD in plant [59]. Except for *GhCYP82D*, *GbTSA1/GbTSB1*, *GhLMMD* and *GhSSI2s* [47, 60–62], few lesion mimic phenotypes have been found in allotetraploid cotton. In this study, we found that inhibiting the expression of cotton ACLB would induce a significant cell death phenotype. This discovery provides a potential gene for the study of cotton LMMs.

Salicylic acid (SA) plays a key role in the defense responses against biotrophic and hemi-biotrophic pathogens [63]. There are also reports suggesting roles for SA in osmotic responses [64]. Plants accumulate SA in the infected site and the distal leaves to response pathogen attack [65]. In our study, when ACLB gene was knocked down, the SA signalling pathway-related genes and PR genes were activated. This finding suggests that the immune response pathway negatively regulated by ACLB may be related to the SA signaling pathway. In addition, our results further prove that senescence-related transcription factors were activated and expressed in ACLB-silenced plants, and profuse H_2_O_2_ was accumulated in newly-grown leaves and stem apexes.

In *Arabidopsis*, mutants with reduced ACL activity exhibited excessive accumulation of anthocyanin and stress mRNA in vegetative tissues [43]. In rice, the *OsACLA-2* mutant is a lesion mimic mutant [44], implying that the ACLA subunit functional divergence during the evolution of plants. In our study, the *Arabidopsis* mutant *aclb-2* did not produce lesion mimic phenotype, suggesting that mutation of a single gene synthesizing ACLB subunits would not induce macroscopic cell death. In cotton, simultaneously silencing ACLB-1 and ACLB-2 induced the cell death of newly-grown leaves and stem apexes either in TM-1 or Hai7124.

In the VIGS assay, ACLB-silenced induced strong cell death in cotton, which led to the failure of subsequent fungal pathogenicity assays. However, it is impossible to guarantee silencing specificity, because of the high homology of the homoeologous genes in the cotton genome (Table S3), which poses a challenge for the application of ACLB-2 gene in cotton engineering disease resistance. Given that *aclb-2* mutant did not induce macroscopic cell death and exhibited the increasing disease resistance, which provides new insights into the applications of ACLB-2 in cotton. It would be worth investigating whether a single *ACLB-2* gene mutants via CRISPR in cotton improve disease resistance without causing growth inhibition.

## Conclusion

Our results prove that the ACLB subunit seems to have a new function of negatively affecting cell death in cotton. In addition, *aclb-2* showed enhanced resistance to *V. dahliae*, indicating that reducing the expression of *AtACLB-2* alone could also release a suppressed immune response. Taken together, our findings could provide new insights into the application of cotton ACLB-2 gene in resistance to VW.

## Methods

### Plant materials and pathogen strain

Seeds of *G. hirsutum* acc. TM-1 (cultivar susceptible to *Verticillium dahliae*) and *G. barbadense* acc. Hai7124 (cultivar resistant to *V. dahliae*) were harvested from our institute (Nanjing, Jiangsu province, China) and grown in the controlled climate chamber (under lighting 16 h/28 °C, darkness 8 h/25 °C). Hai7124 (M210054; Jiangsu, China) and TM-1 (ZM-50380) were originally collected from the National Medium-term Gene Bank of Cotton in China. *G. australe* (I-62510), a native Australian wild species being highly resistant to *V. dahliae*, was kindly provided by Dr. Kunbo Wang, professor of Cotton Research Institute of Chinese Academy of Agricultural Sciences and used for gene cloning. Wild-type *Arabidopsis* (Columbia ecotype, Col-0) and *aclb-2* mutant (annotated locus: AT5G49460, *aclb-2*, T-DNA identifier: SALK_138734 bought from the Arabidopsis Biological Resource Center at Ohio State University) were grown in a growth chamber under a 16/8 h light/dark cycle at a constant temperature of 22 °C. All the above plant materials, including *G. hirsutum* acc. TM-1, *G. barbadense* acc. Hai7124, *G. australe,* Wild-type *Arabidopsis* (Columbia ecotype, Col-0) and *aclb-2* mutant are deposited at Cotton Research Institute of Nanjing Agricultural University.

*V. dahliae* wild-type strain V991 (highly virulent and defoliating strain), kindly provided by Prof. Ling Lin, Institute of Plant Protection of Jiangsu Academy of Agricultural Sciences of China, was cultured on potato dextrose agar (PDA) or in liquid Czapek medium at 25 °C. The preparation of conidial suspensions (10^7^ conidia mL^− 1^) and inoculation were performed as standard inoculation procedures [66]. Control plants were inoculated with sterile water.

### Quantitative RT-PCR and phylogenetic analysis

Total RNA was isolated from fresh plant tissue using the MolPure® Plant Plus RNA Kit (Shanghai Yeasen Biotechnology Co., Ltd., Shanghai, China). And then, first-strand cDNA was generated from 2 μg of total RNA using the Hifair® II 1st Strand cDNA Synthesis Kit (gDNA digester plus) (Shanghai Yeasen Biotechnology Co., Ltd., Shanghai, China). Hieff qPCR SYBR Green Master Mix (Yeasen Biotech) and the LightCycler 480 System (Roche) were used for the qRT-PCR assays. Relative expression levels were calculated according to the 2^–ΔCT^ [2^-(CT genes of interest - CT internal controls)^] method [67] and the cotton Histone3 gene (AF024716) and *Arabidopsis* Ubq5 (At3g62250) were individually used as internal controls. The full-length cDNA sequences of genes were amplified using the clone primers. All primers used were synthesized by Tsingke Biotech and the full sequences are disclosed in Table S4.

The seed sequences of CoA_ligase (PF00549) and Citrate_synthase (PF00285) from Pfam (31st edition, http://pfam.xfam.org) were download to search for ATP-citrate lyases in the predicted proteome from 22 species by BLASTp [68]. Predicted conserved domains were determined using SMART (http://smart.embl-heidelberg.de). Sequences containing both CoA_ligase and Citrate_synthase functional domains were selected as ACLB homologues from each plant species. The phylogenetic tree was generated with MEGA v. 7.0 software (http://megasoftware.net) using the maximum likelihood method with 1000 bootstrap replications.

### Gene expression analysis

The genome and Illumina RNA-seq data of *G. hirsutum* acc. TM-1 were available at the Sequence Read Archive (SRA) under accession number PRJNA248163 [69]. The genome and Illumina RNA-seq data of *G. barbadense* cv. Hai7124 were available at the SRA under accession number PRJNA490626 [70]. The gene expression analysis was performed as previously reported [70].

### Subcellular localization

The ORFs of *GausACLB-2* amplified from *G. australe* were fused in GFP into a binary vector pBIN-GFP4. AtPIP2A fusion RFP protein was used as plasma membrane marker [71]. The excitation wavelengths for imaging GFP and RFP fusions were 488 and 580 nm, respectively. The recombinant vector was then transformed into tobacco (*N. benthamiana*) leaf epidermal cells as described by Lu et al. [72]. Fluorescent signals were recorded and visualized by using a Leica TCS SP8 SR Laser scanning confocal microscope (Leica, Germany) with a 488 nm or 580 nm laser, and photographed with a LEICA DFC420 camera under 20X objective lenses. Images were acquired at 1024 × 1024 resolution using LAS X software (Leica Application Suite X) and processed using Adobe Illustrator.

### Virus-induced gene silencing (VIGS) procedure

The conserved regions of ACLB were amplified from the cDNA of cv. TM-1 and cv. Hai7124 using the corresponding primer pairs. The conserved region fragments were then cloned into the tobacco rattle virus (TRV) binary vector pTRV2 between the BamHI and SacI sites, constructing vectors were named as TRV: *GhACLB* or TRV: *GbACLB*. These vectors were introduced into *Agrobacterium tumefaciens* GV3101 by freeze-thaw method. Strains containing different pTRV2 vectors and pTRV1 strains were mixed in a 1:1 ratio (v: v). *Cloroplastos alterados* 1 (CLA1) gene served as a positive marker for evaluating VIGS efficiency. The repeated VIGS experiment determined the time point when the plants carrying TRV: *CLA* showed albino phenotype as 0 h (the inoculation time on the day). A total of 7 time-points, namely, − 48 h, − 24 h, 0 h, 3 h, 6 h, 12 h and 24 h, were selected to extract RNA from newly-grown leaves to examine the expression of target genes. The experiments were repeated three times independently with more than 30 plants used for each treatment.

### 3,3′-diaminobenzidine (DAB) staining and determination of H_2_O_2_ content

The seedlings of TM-1 and Hai7124 were treated with VIGS, and newly-grown leaves were sampled at the aforementioned seven time-points. The cotton leaves were incubated in staining solution (1 mg L^− 1^ DAB, 10 mmol L^− 1^ sodium phosphate at pH 7.0, and 0.05% v/v Tween-20) under gentle vacuum in darkness at 25 °C for 3 h, decolorized with 95% ethanol until the green color faded to a yellowish color, and observed under a stereomicroscope. Sample the leaves of at least five plants for each treatment.

The fresh leaves were quickly ground into powder in liquid nitrogen, and 1.0 g of power was accurately weighed for subsequent quantitative analysis. The weighed sample was extracted in 1.0 mL of 0.1 mol L^− 1^ potassium phosphate buffer (pH 7.0), centrifuged at 10,000×g at 4 °C for 5 minutes, and then, the supernatant was used for H_2_O_2_ quantification. The levels of H_2_O_2_ in plant tissues were determined using the spectrophotometric method with an H_2_O_2_ assay kit (Nanjing Jiancheng, China).

### Generation of transgenic *Arabidopsis*

The full-length of 1827 bp *GausACLB-2* coding sequence was cloned and then inserted into the pBI121 (Cambia) plant binary vector containing a kanamycin resistance gene with the In-Fusion HD Cloning Plus (Clontech). This vector was transformed into *Agrobacterium tumefaciens* GV3101 using the freeze–thaw method. *A. thaliana* Col-0 (wild type) and *aclb-2* (mutant) were transformed with the overexpression vector via the floral dip method [73]. T_0_–T_3_ transgenic seeds were then spread evenly on plates of MS medium (50 μM kanamycin) to select for positive transformants. T_3_ homozygous plants were screened by three successive generations, and were used for *GausACLB-2* gene expression analysis using qRT-PCR. The *Arabidopsis AtActin* gene (At1g49240) was selected as an internal control. Independent qRT-PCR experiments were performed in three biological replicates. T_3_ transgenic lines were used in subsequent experiments. From 7 d post inoculation (dpi) with *V. dahliae*, the symptoms of *Arabidopsis* plants were investigated daily and the rate of diseased leaves was recorded. The degree of VW resistance was graded from 0 to 4 according to the extent of leaf chlorosis. The disease index (DI) was calculated as follows: DI = [(∑ disease grades × number of infected plants) / (total number of scored plants × 4)] × 100.

### Fungal recovery assay and biomass quantification

For fungal recovery assay in *Arabidopsis* as described previously in cotton [55], the seedlings were treated with V991 for 10 days, and then stems with the length of 2.0 cm above the base were cut off and sterilized shortly by alcohol and placed on a PDA medium for 3 days and then photographed.

For biomass quantification in planta, on 14 dpi with V991, DNA from multiple tissues of plants were extracted using a Plant DNA Mini Kit (Aidlab Biotechnologies, Beijing, China). The internal transcribed spacer (ITS) region of ribosomal DNA and *V. dahliae*-specific reverse primer STVe1-R were used to quantify the biomass of *V. dahliae*, and *Atubq5* was used as reference genes for the normalization of quantitative reverse transcription PCR (qRT-PCR) data.

### ACL activity and citrate assays

The ACL activity was determined as previously described with minor modifications [36]. In brief, the assay detects the ACL-catalyzed generation of oxaloacetate by coupling the oxidation of NADH catalyzed by malate dehydrogenase. The oxidation of NADH was monitored by the change in 340 nm light absorption, and ACL activity was calculated using the molar extinction coefficient of NADH (6.22 mM^− 1^ cm^− 1^). The ACL assay was conducted in total volume of 1 mL, containing 200 μL of extract, 20 mm MgCl_2_, 200 mM Tris-HCl (pH 8.4), 10 mM ATP, 1 mM DTT, 10 mM citrate, 0.2 mM CoA, 0.1 mM NADH and 6 units of malate dehydrogenase. Citrate were detected using citric acid (CA) content assay kit (Solarbio® BC2150).

## Supplementary Information

**Additional file 1: Fig. S1.** Primary structure analysis of *GausACLB-2*. (a) Gene structures of the *AtACLB-1*, *AtACLB-2*, *GausACLB-1* and *GausACLB-2*. Black rectangles represent exons. (b) Protein sequence alignment of ACLB-2 genes. PF00549, CoA_ligase; PF00285, Citrate_synthase.

**Additional file 2: Fig. S2.** Cis- acting elements analysis of *GausACLB-2* promoter. (a) The putative transcription start site of *GausACLB-2* promoter. (b) The *cis*-acting element of the *GausACLB-1* promoter.

**Additional file 3: Fig. S3.** The relative expression levels of ACLB-2 in the roots and leaves of TM-1 (*G. hirsutum*), H7124 (*G. barbadense*) and *G. australe*. Data are shown as mean ± SE of three biological replicates.

**Additional file 4: Fig. S4.** Relative expression levels of senescence-related genes were markedly enhanced in knockdown ACLB plants. (a) Relative expression levels of senescence-related genes in control and VIGS-TM-1 plants. (b) Relative expression levels of senescence-related genes in control and VIGS-Hai7124 plants. Data are shown as mean ± SE of three biological replicates (*P < 0.05, **P < 0.01, Student’s *t*-test).

**Additional file 5: Fig. S5.** Validation of *Arabidopsis* (AT5G49460, *aclb-2*) mutants. (a) Schematic diagram showing two pairs of primers to PCR for validating T-DNA insertion. (b) The results of PCR amplification of Col-0 and *aclb-2* mutants. (c) The relative expression level of *AtACLB-2* in Col-0 and *aclb-2*. Data are shown as mean ± SE of three biological replicates (**P < 0.01, Student’s *t*-test). (d) The 14-days-old seedlings of the Col-0 grew obviously faster than that of *aclb-2*. Scale bars, 1 cm. (e) Root of Col-0 was obviously longer than that of *aclb-2*. Data are shown as mean ± SE of six biological replicates (**P < 0.01, Student’s *t*-test).

**Additional file 6: Table S1.** Distribution and name of ATP citrate lyase in cotton.

**Additional file 7: Table S2.** *Cis*-element prediction of upstream sequence of *GausACLB-2* gene.

**Additional file 8: Table S3.** Comparison of homology between ACLB protein in *G. australe*, *G. hirsutum* and *G. barbadense*.

**Additional file 9: Table S4.** Primers used in this study.

**Additional file 10: Data S1.** Amino acid sequences of ACL proteins in 22 plant species.

## Data Availability

All data supporting the conclusions of this article are included in this manuscript and its supplementary information files. The genome and Illumina RNA-seq data of *G. hirsutum* acc. TM-1 and *G. barbadense* acc. Hai7124 were available at the NCBI Sequence Read Archive under accession number PRJNA490626 and PRJNA248163, respectively.

## Abbreviations

ACL: ATP-citrate lyase
PCD: Programmed cell death
PAMP: Pathogen-associated molecular pattern
ETI: Effector-triggered immunity
HR: Hypersensitive responses
NLR: Nucleotide-binding leucine-rich repeat
ZAR1: HOPZ-ACTIVATED RESISTANCE 1
ZRK1: HOPZ-ETI-DEFICIENT 1-related kinases
WAKs: Wall-associated protein kinases
LMMs: Lesion mimics mutants
Mya: Million years ago
SCSα: Succinyl-CoA synthase
CS: Citrate synthase
VIGS: Virus-induced gene silencing
VW: Verticillium wilt
TSS: Transcription start site
qRT-PCR: Quantitative reverse transcription PCR
ROS: Reactive oxygen species
DAB: 3, 3′-diaminobenzidine
WT: Wild-type
OE: Overexpressing
CoA: Acetyl-CoA
HATs: Histone acetyl transferases
SA: Salicylic acid
PDA: Potato dextrose agar medium
SRA: Sequence Read Archive
TRV: Tobacco rattle virus
CLA1: *Cloroplastos alterados* 1
DI: Disease index
ITS: Internal transcribed spacer
CA: Citric acid

## References

[1] Van Hautegem T, Waters AJ, Goodrich J, Nowack MK (2015). Only in dying, life: programmed cell death during plant development. Trends Plant Sci.

[2] Dickman M, Williams B, Li Y, De Figueiredo P, Wolpert T (2017). Reassessing apoptosis in plants. Nat Plants.

[3] Kabbage M, Kessens R, Bartholomay LC, Williams B (2017). The life and death of a plant cell. Annu Rev Plant Biol.

[4] Kuriyama H, Fukuda H (2002). Developmental programmed cell death in plants. Curr Opin Plant Biol.

[5] Locato V, De Gara L (2018). Programmed cell death in plants: an overview. Methods Mol Biol.

[6] Akira S, Uematsu S, Takeuchi O (2006). Pathogen recognition and innate immunity. Cell..

[7] Jones JDG, Dangl JL (2006). The plant immune system. Nature..

[8] Zhou J-M, Zhang Y (2020). Plant immunity: danger perception and signaling. Cell..

[9] Hammond-Kosack KE, Jones JD (1996). Resistance gene-dependent plant defense responses. Plant Cell.

[10] Muthamilarasan M, Prasad M (2013). Plant innate immunity: an updated insight into defense mechanism. J Biosci.

[11] Wang J, Wang J, Hu M (2019). Ligand-triggered allosteric ADP release primes a plant NLR complex. Science..

[12] Wang J, Hu M, Wang J, Qi J, Han Z, Wang G, Qi Y, Wang HW, Zhou JM, Chai J (2019). Reconstitution and structure of a plant NLR resistosome conferring immunity. Science..

[13] Bi G, Su M, Li N (2021). The ZAR1 resistosome is a calcium-permeable channel triggering plant immune signaling. Cell..

[14] Gong Z, Qi J, Hu M, Bi G, Zhou J-M, Han G-Z (2022). The origin and evolution of a plant resistosome. Plant Cell.

[15] Wu C, Bordeos A, Madamba MR (2008). Rice lesion mimic mutants with enhanced resistance to diseases. Mol Gen Genomics.

[16] Lorrain S, Vailleau F, Balagué C, Roby D (2003). Lesion mimic mutants: keys for deciphering cell death and defense pathways in plants?. Trends Plant Sci.

[17] Quesada V, Sarmiento-Mañús R, González-Bayón R, Hricová A, Ponce MR, Micol JL (2013). Porphobilinogen deaminase deficiency alters vegetative and reproductive development and causes lesions in *Arabidopsis*. PLoS One.

[18] Walbot V, Hoisington DA, Neuffer MG (1983). Disease lesion mimic mutations. Sci China Life Sci.

[19] Hu G, Yalpani N, Briggs SP, Johal GS (1998). A porphyrin pathway impairment is responsible for the phenotype of a dominant disease lesion mimic mutant of maize. Plant Cell.

[20] Wolter M, Hollricher K, Salamini F, Schulze-Lefert P (1993). The mlo resistance alleles to powdery mildew infection in barley trigger a developmentally controlled defence mimic phenotype. Mol Gen Genomics.

[21] Nair SK, Tomar SMS (2001). Genetical and anatomical analyses of a leaf flecking mutant in *Triticum aestivum* L. Euphytica..

[22] Wang F, Wu W, Wang D (2016). Characterization and genetic analysis of a novel light-dependent lesion mimic mutant, lm3, showing adult-plant resistance to powdery mildew in common wheat. PLoS One.

[23] Yin Z, Chen J, Zeng L (2000). Characterizing rice lesion mimic mutants and identifying a mutant with broad-spectrum resistance to rice blast and bacterial blight. Mol Plant Microbe In.

[24] Sun C, Liu L, Tang J (2011). RLIN1, encoding a putative coproporphyrinogen III oxidase, is involved in lesion initiation in rice. J Genet Genom.

[25] Dietrich RA, Richberg MH, Schmidt R, Dean C, Dangl JL (1997). A novel zinc finger protein is encoded by the *Arabidopsis* LSD1 gene and functions as a negative regulator of plant cell death. Cell..

[26] Büschges R, Hollricher K, Panstruga R (1997). The barley Mlo gene: a novel control element of plant pathogen resistance. Cell..

[27] Kachroo P, Shanklin J, Shah J, Whittle EJ, Klessig DF (2001). A fatty acid desaturase modulates the activation of defense signaling pathways in plants. Proc Natl Acad Scie U S A.

[28] Zeng LR, Qu S, Bordeos A (2004). Spotted leaf11, a negative regulator of plant cell death and defense, encodes a U-box/armadillo repeat protein endowed with E3 ubiquitin ligase activity. Plant Cell.

[29] Qiao Y, Jiang W, Lee J (2010). SPL28 encodes a clathrin-associated adaptor protein complex 1, medium subunit micro 1 (AP1M1) and is responsible for spotted leaf and early senescence in rice (*Oryza sativa*). New Phytol.

[30] Chen X, Hao L, Pan J (2012). SPL5, a cell death and defense-related gene, encodes a putative splicing factor 3b subunit 3 (SF3b3) in rice. Mol Breed.

[31] Wang Z, Wang Y, Hong X (2015). Functional inactivation of UDP-N-acetylglucosamine pyrophosphorylase 1 (UAP1) induces early leaf senescence and defence responses in rice. J Exp Bot.

[32] Zhu X, Yin J, Liang S (2016). The multivesicular bodies (MVBs)-localized AAA ATPase LRD6-6 inhibits immunity and cell death likely through regulating MVBs-mediated vesicular trafficking in rice. PLoS Genet.

[33] Liu Q, Ning Y, Zhang Y (2017). OsCUL3a negatively regulates cell death and immunity by degrading *OsNPR1* in rice. Plant Cell.

[34] Wang S, Lei C, Wang J (2017). SPL33, encoding an eEF1A-like protein, negatively regulates cell death and defense responses in rice. J Exp Bot.

[35] Sun L, Wang Y, Liu LL (2017). Isolation and characterization of a spotted leaf 32 mutant with early leaf senescence and enhanced defense response in rice. Sci Rep.

[36] Fatland BL, Ke J, Anderson MD (2002). Molecular characterization of a heteromeric ATP-citrate lyase that generates cytosolic acetyl-coenzyme a in Arabidopsis. Plant Physiol.

[37] Elshourbagy NA, Near JC, Kmetz PJ (1992). Cloning and expression of a human ATP-citrate lyase cDNA. Eur J Biochem.

[38] Hu XM, Shi CY, Liu X, Jin LF, Liu YZ, Peng SA (2015). Genome-wide identification of citrus ATP-citrate lyase genes and their transcript analysis in fruits reveals their possible role in citrate utilization. Mol Gen Genomics.

[39] Chypre M, Zaidi N, Smans K (2012). ATP-citrate lyase: a mini-review. Biochem Biophys Res Commun.

[40] Zaidi N, Swinnen JV, Smans K (2012). ATP-citrate lyase: a key player in cancer metabolism. Cancer Res.

[41] Hatzivassiliou G, Zhao F, Bauer DE (2005). ATP citrate lyase inhibition can suppress tumor cell growth. Cancer Cell.

[42] Wellen KE, Hatzivassiliou G, Sachdeva UM, Bui TV, Cross JR, Thompson CB (2009). ATP-citrate lyase links cellular metabolism to histone acetylation. Science..

[43] Fatland BL, Nikolau BJ, Wurtele ES (2005). Reverse genetic characterization of cytosolic acetyl-CoA generation by ATP-citrate lyase in *Arabidopsis*. Plant Cell.

[44] Ruan B, Hua Z, Zhao J (2019). OsACL-A2 negatively regulates cell death and disease resistance in rice. Plant Biotechnol J.

[45] Webber JM (1936). Cytogenetic notes on cotton and cotton relatives. II. Science.

[46] Senchina DS, Alvarez I, Cronn RC (2003). Rate variation among nuclear genes and the age of polyploidy in Gossypium. Mol Biol Evol.

[47] Wendel JF, Olson PD, Stewart JM (1989). Genetic diversity, introgression, and independent domestication of old world cultivated cottons. Am J Bot.

[48] Wendel JF, Cronn RC (2003). Polyploidy and the evolutionary history of cotton. Adv Agron.

[49] Reese MG (2001). Application of a time-delay neural network to promoter annotation in the Drosophila melanogaster genome. Comput Chem Eng.

[50] Lescot M, Déhais P, Thijs G, Marchal K, Moreau Y, Van de Peer Y, Rouzé P, Rombauts S (2002). PlantCARE, a database of plant cis-acting regulatory elements and a portal to tools for in silico analysis of promoter sequences. Nucleic Acids Res.

[51] Mo S, Zhang Y, Wang X (2021). Cotton *GhSSI2* isoforms from the stearoyl acyl carrier protein fatty acid desaturase family regulate Verticillium wilt resistance. Mol Plant Pathol.

[52] Pietrocola F, Galluzzi L, Bravo-San Pedro JM, Madeo F, Kroemer G (2015). Acetyl coenzyme a: a central metabolite and second messenger. Cell Metab.

[53] Tumaney AW, Ohlrogge JB, Pollard M (2004). Acetyl coenzyme a concentrations in plant tissues. J Plant Physiol.

[54] Brodersen P, Petersen M, Pike HM (2002). Knockout of *Arabidopsis* accelerated-cell-death11 encoding a sphingosine transfer protein causes activation of programmed cell death and defense. Genes Dev.

[55] Pitsili E, Phukan UJ, Coll NS (2020). Cell death in plant immunity. Cold Spring Harb Perspect Biol.

[56] Bozhkov PV, Lam E (2011). Green death: revealing programmed cell death in plants. Cell Death Dis.

[57] Fuchs Y, Steller H (2011). Programmed cell death in animal development and disease. Cell..

[58] Teng X, Cheng WC, Qi B (2011). Gene-dependent cell death in yeast. Cell Death Dis.

[59] Bruggeman Q, Raynaud C, Benhamed M, Delarue M (2015). To die or not to die? Lessons from lesion mimic mutants. Front Plant Sci.

[60] Sun L, Zhu L, Xu L, Yuan D, Min L, Zhang X (2014). Cotton cytochrome P450 CYP82D regulates systemic cell death by modulating the octadecanoid pathway. Nat Commun.

[61] Chai Q, Shang X, Wu S (2017). 5-Aminolevulinic acid Dehydratase gene dosage affects programmed cell death and immunity. Plant Physiol.

[62] Miao Y, Xu L, He X (2019). Suppression of tryptophan synthase activates cotton immunity by triggering cell death via promoting SA synthesis. Plant J.

[63] Vlot AC, Dempsey DA, Klessig DF (2009). Salicylic acid, a multifaceted hormone to combat disease. Annu Rev Phytopathol.

[64] Borsani O, Valpuesta V, Botella MA (2001). Evidence for a role of salicylic acid in the oxidative damage generated by NaCl and osmotic stress in *Arabidopsis* seedlings. Plant Physiol.

[65] Jayakannan M, Bose J, Babourina O, Rengel Z, Shabala S (2015). Salicylic acid in plant salinity stress signalling and tolerance. Plant Growth Regul.

[66] Wang G, Xu J, Li L (2020). GbCYP86A1-1 from *Gossypium barbadense* positively regulates defence against *Verticillium dahliae* by cell wall modification and activation of immune pathways. Plant Biotechnol J.

[67] Schmittgen TD, Livak KJ (2008). Analyzing real-time PCR data by the comparative C(T) method. Nat Protoc.

[68] Altschul SF, Gish W, Miller W (1990). Lipman, basic local alignment search tool. J Mol Biol.

[69] Zhang T, Hu Y, Jiang W (2015). Sequencing of allotetraploid cotton (*Gossypium hirsutum* L. acc. TM-1) provides a resource for fiber improvement. Nat Biotechnol.

[70] Hu Y, Chen J, Fang L (2019). *Gossypium barbadense* and *Gossypium hirsutum* genomes provide insights into the origin and evolution of allotetraploid cotton. Nat Genet.

[71] Cutler SR, Ehrhardt DW, Griffitts JS, Somerville CR (2000). Random GFP::cDNA fusions enable visualization of subcellular structures in cells of *Arabidopsis* at a high frequency. Proc Natl Acad Sci U S A.

[72] Lu S, Zhu T, Wang Z (2021). *Arabidopsis* immune-associated nucleotide-binding genes repress heat tolerance at the reproductive stage by inhibiting the unfolded protein response and promoting cell death. Mol Plant.

[73] Clough SJ, Bent AF (1998). Floral dip: a simplified method for *Agrobacterium*-mediated transformation of *Arabidopsis thaliana*. Plant J.

