# Supplementary figures and images for: ATP-citrate lyase B (ACLB) negatively affects cell death and resistance to Verticillium wilt
Source: BMC Plant Biol. 2022 Sep 16;22:443. doi: 10.1186/s12870-022-03834-z (PMC9479425; doi:10.1186/s12870-022-03834-z)

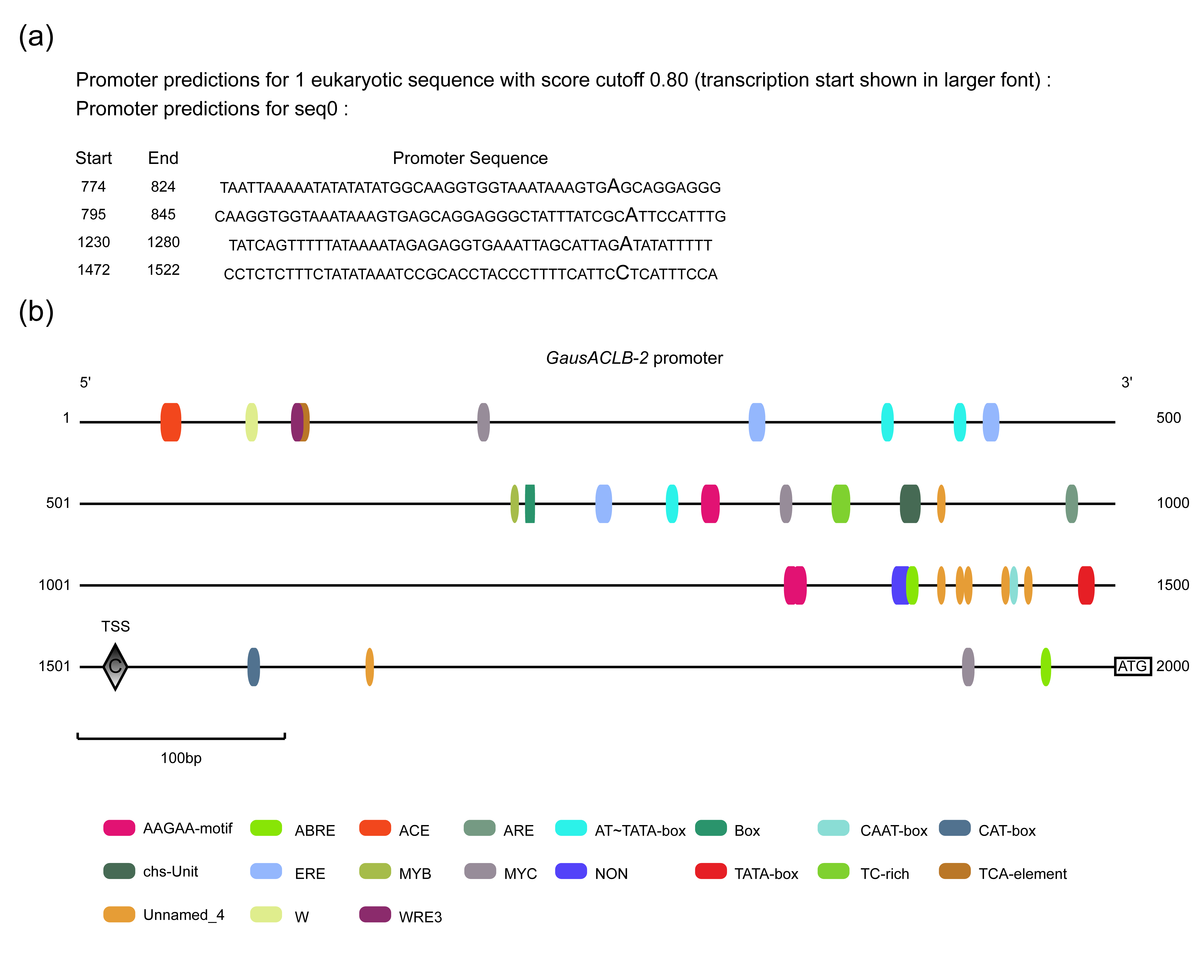

Supplement: Supplementary file 2 — Additional file 2: Fig. S2. Cis- acting elements analysis of GausACLB-2 promoter. (a) The putative transcription start site of GausACLB-2 promoter. (b) The cis-acting element of the GausACLB-1 promoter. [file 12870_2022_3834_MOESM2_ESM.tif]

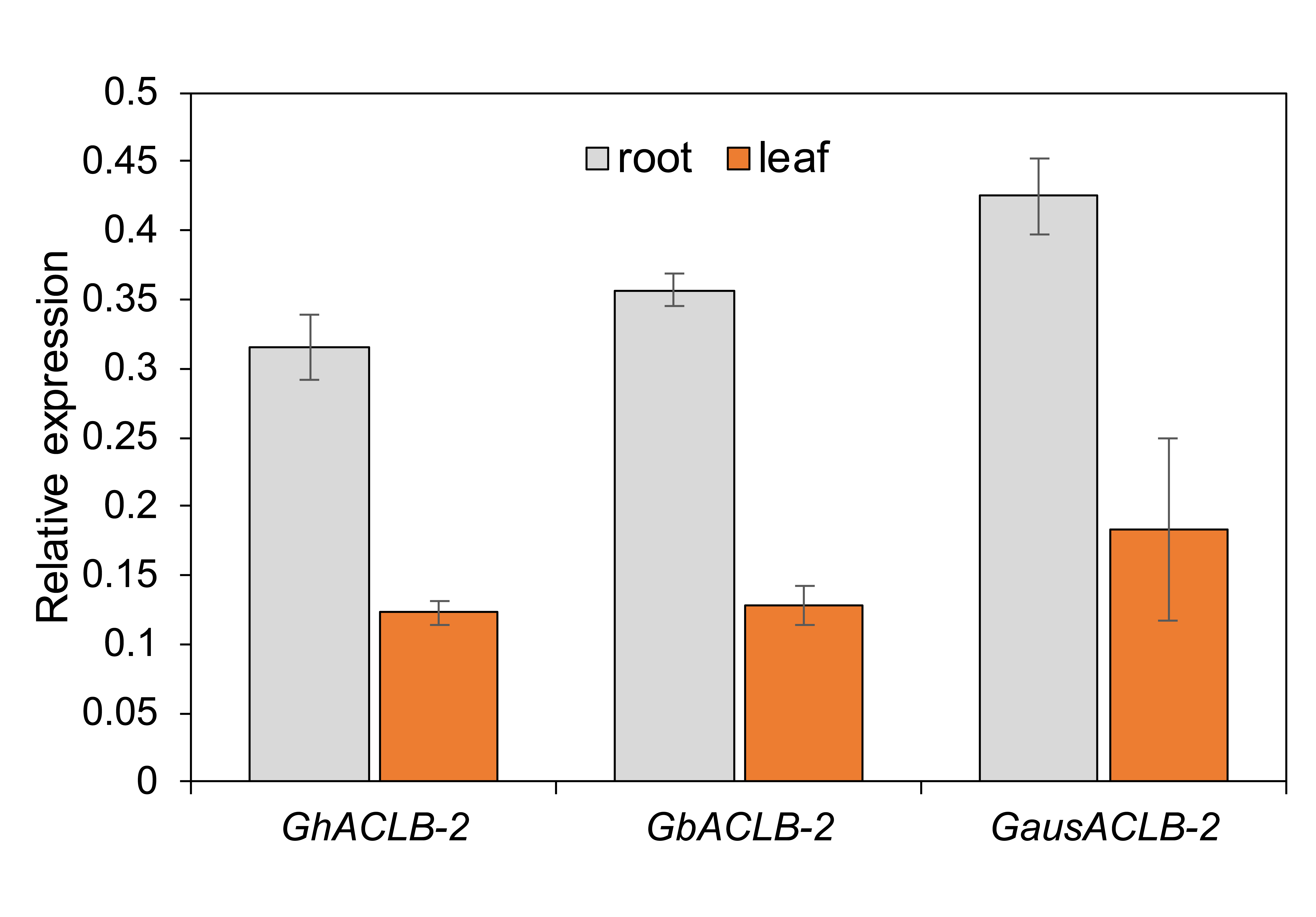

Supplement: Supplementary file 3 — Additional file 3: Fig. S3. The relative expression levels of ACLB-2 in the roots and leaves of TM-1 (G. hirsutum), H7124 (G. barbadense) and G. australe. Data are shown as mean ± SE of three biological replicates. [file 12870_2022_3834_MOESM3_ESM.tif]

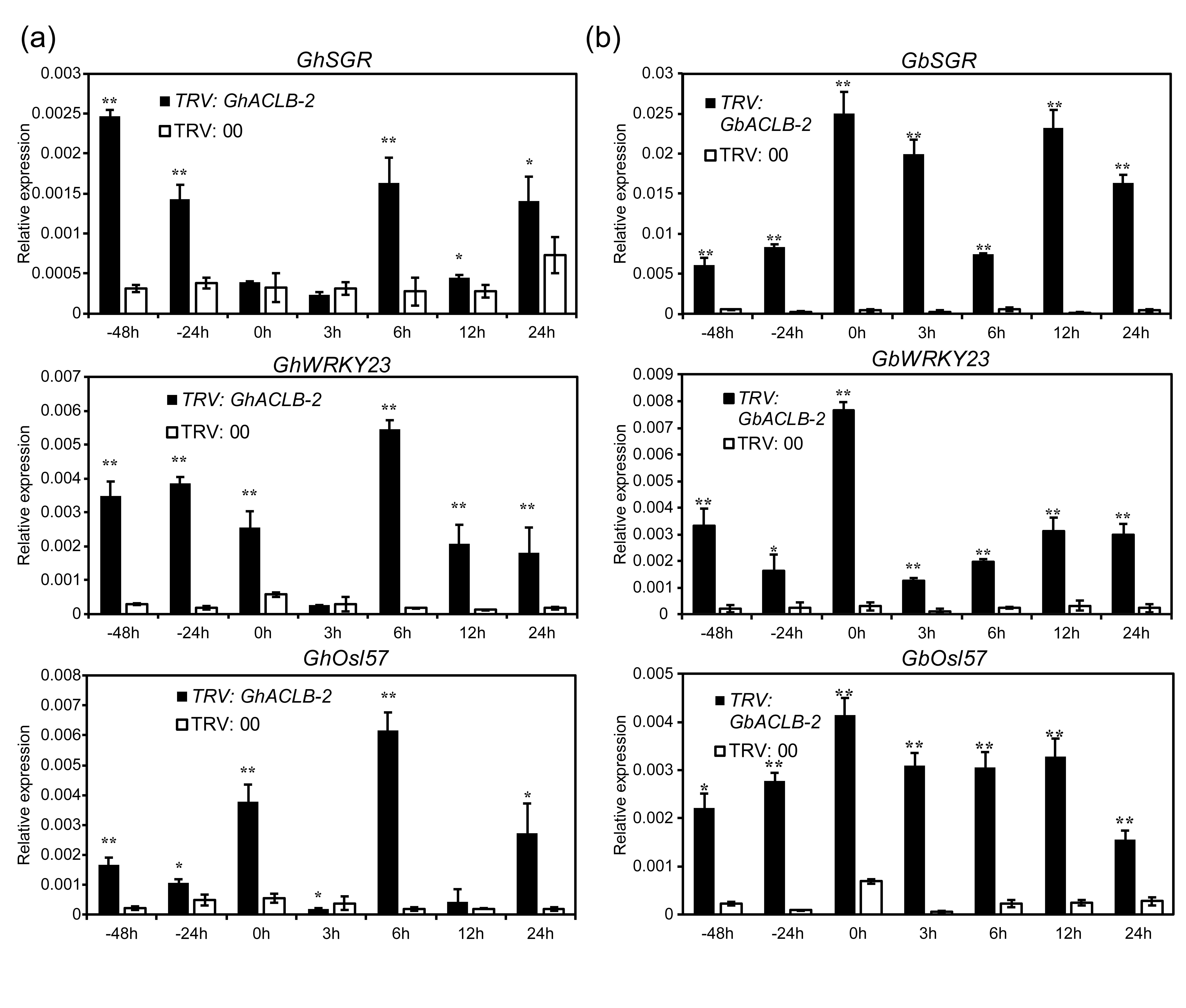

Supplement: Supplementary file 4 — Additional file 4: Fig. S4. Relative expression levels of senescence-related genes were markedly enhanced in knockdown ACLB plants. (a) Relative expression levels of senescence-related genes in control and VIGS-TM-1 plants. (b) Relative expression levels of senescence-related genes in control and VIGS-Hai7124 plants. Data are shown as mean ± SE of three biological replicates (*P < 0.05, **P < 0.01, Student’s t-test). [file 12870_2022_3834_MOESM4_ESM.tif]

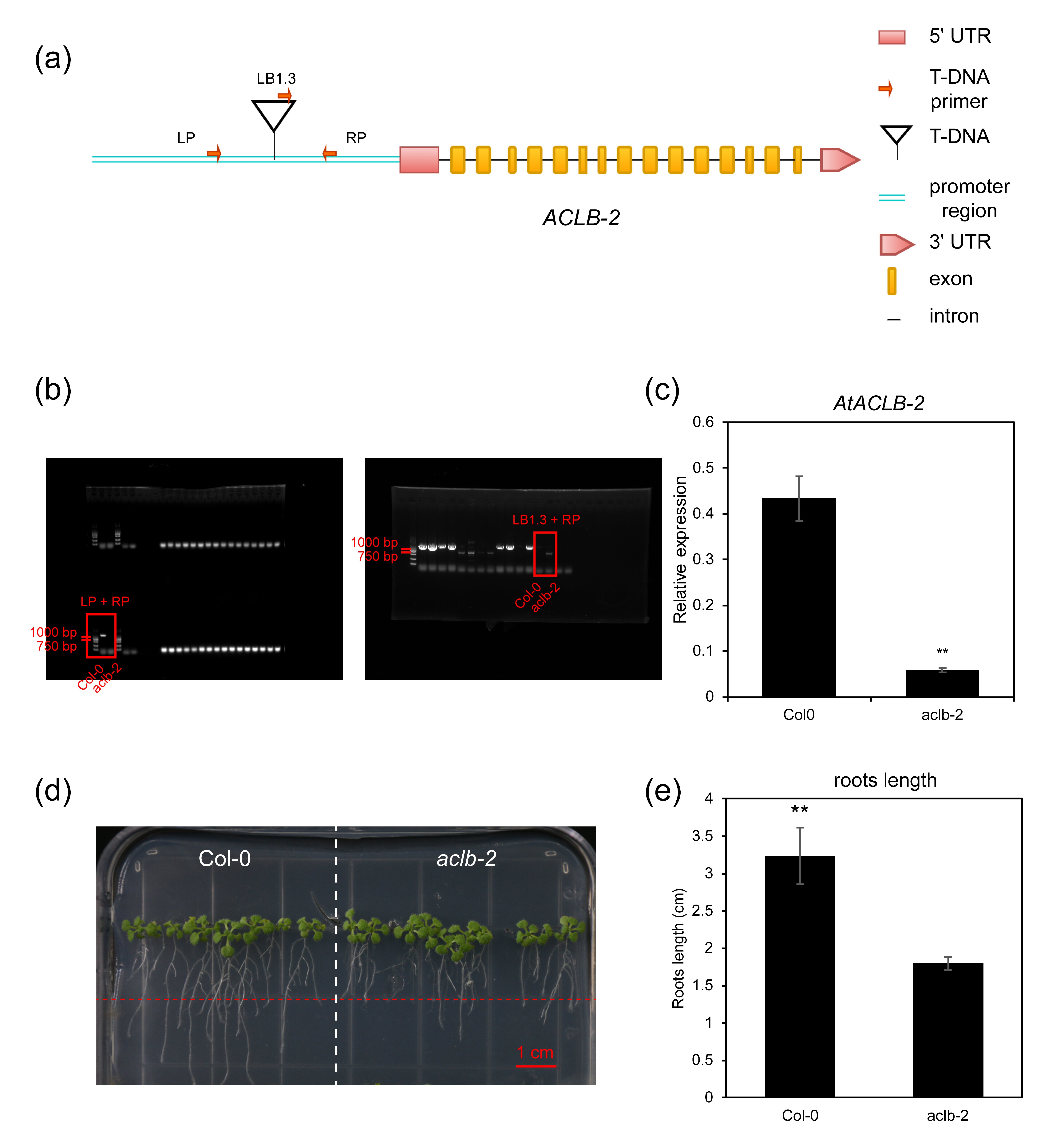

Supplement: Supplementary file 5 — Additional file 5: Fig. S5. Validation of Arabidopsis (AT5G49460, aclb-2) mutants. (a) Schematic diagram showing two pairs of primers to PCR for validating T-DNA insertion. (b) The results of PCR amplification of Col-0 and aclb-2 mutants. (c) The relative expression level of AtACLB-2 in Col-0 and aclb-2. Data are shown as mean ± SE of three biological replicates (**P < 0.01, Student’s t-test). (d) The 14-days-old seedlings of the Col-0 grew obviously faster than that of aclb-2. Scale bars, 1 cm. (e) Root of Col-0 was obviously longer than that of aclb-2. Data are shown as mean ± SE of six biological replicates (**P < 0.01, Student’s t-test). [file 12870_2022_3834_MOESM5_ESM.jpg]
