# Supplementary material for: ATP-citrate lyase B (ACLB) negatively affects cell death and resistance to Verticillium wilt
Source: BMC Plant Biol. 2022 Sep 16;22:443. doi: 10.1186/s12870-022-03834-z (PMC9479425; doi:10.1186/s12870-022-03834-z)
Supplement: Supplementary file 6 — Additional file 6: Table S1. Distribution and name of ATP citrate lyase in cotton. [file 12870_2022_3834_MOESM6_ESM.docx]

|  | Names | **Gene ID** | Names | **Gene ID** |
| --- | --- | --- | --- | --- |
| *G. hirsutum* | *GhACLB-1A* | *GH_A01G0298* | *GhACLA-1A* | *GH_A04G1649* |
|  | *GhACLB-1D* | *GH_D01G0282* | *GhACLA-1D* | *GH_D04G1999* |
|  | *GhACLB-2A* | *GH_A10G1881* | *GhACLA-2A* | *GH_A11G3084* |
|  | *GhACLB-2D* | *GH_D10G1973* | *GhACLA-2D* | *GH_D11G3112* |
|  |  |  | *GhACLA-3A* | *GH_A12G1634* |
|  |  |  | *GhACLA-3D* | *GH_D12G1641* |
| *G. barbadense* | *GbACLB-1A* | *GB_A01G0289* | *GbACLA-1A* | *GB_A04G1694* |
|  | *GbACLB-1D* | *GB_D01G0288* | *GbACLA-1D* | *GB_D04G2087* |
|  | *GbACLB-2A* | *GB_A10G2003* | *GbACLA-2A* | *GB_A11G3154* |
|  | *GbACLB-2D* | *GB_D10G1989* | *GbACLA-2D* | *GB_D11G3151* |
|  |  |  | *GbACLA-3A* | *GB_A12G1707* |
|  |  |  | *GbACLA-3D* | *GB_D12G1700* |
| *G. australe* | *GausACLB-1* | *gb\|KAA3467895.1\|* | *GausACLA-1* | *gb\|KAA3462635.1\|* |
|  | *GausACLB-2* | *gb\|KAA3481184.1\|* | *GausACLA-2* | *gb\|KAA3463391.1\|* |
|  |  |  | *GausACLA-3* | *gb\|KAA3472585.1\|* |
| *G. arboreum* | *GaACLB-1A* | *Cotton_A_29624_BGI-A2_v1.0* | *GaACLA-1A* | *Cotton_A_18654_BGI-A2_v1.0* |
|  | *GaACLB-2A* | *Cotton_A_17796_BGI-A2_v1.0* | *GaACLA-2A* | *Cotton_A_23055_BGI-A2_v1.0* |
|  |  |  | *GaACLA-3A* | *Cotton_A_14782_BGI-A2_v1.0* |
| *G. raimondii* | *GrACLB-1D* | *Cotton_D_gene_10015699* | *GrACLA-1D* | *Cotton_D_gene_10034822* |
|  | *GrACLB-2D* | *Cotton_D_gene_10007437* | *GrACLA-2D* | *Cotton_D_gene_10013559* |
|  |  |  | *GrACLA-3D* | *Cotton_D_gene_10012375* |

**Table S1 Distribution and name of ATP citrate lyase in cotton.**
