# Supplementary material for: ATP-citrate lyase B (ACLB) negatively affects cell death and resistance to Verticillium wilt
Source: BMC Plant Biol. 2022 Sep 16;22:443. doi: 10.1186/s12870-022-03834-z (PMC9479425; doi:10.1186/s12870-022-03834-z)
Supplement: Supplementary file 7 — Additional file 7: Table S2. Cis-element prediction of upstream sequence of GausACLB-2 gene. [file 12870_2022_3834_MOESM7_ESM.docx]

| Classified of elements | Cis-acting elements | Sequence | Function |
| --- | --- | --- | --- |
| Basal promoter elements | CAAT-box | CCAAT/CAAAT | common cis-acting element in promoter and enhancer regions |
|  | TATA-box | TACAAAA/TATA | core promoter element around -30 of transcription start |
| Hormones and stress response | TCA-element | CCATCTTTTT | cis-acting element involved in salicylic acid responsiveness |
|  | TC-rich repeats | ATTCTCTAAC | cis-acting element involved in defense and stress responsiveness |
|  | ARE | AAACCA | cis-acting regulatory element essential for the anaerobic induction |
|  | ABRE | ACGTG | cis-acting element involved in the abscisic acid responsiveness |
|  | WUN-motif | AAATTTCCT | wound-responsive element |
| Growth regulation and light response | ACE | CTAACGTATT | cis-acting element involved in light responsiveness |
|  | AT1-motif | AATTATTTTTTATT | part of a light responsive module |
|  | Box 4 | ATTAAT | part of a conserved DNA module involved in light responsiveness |
|  | chs-Unit 1 m1 | ACCTAACCCGG | part of a light responsive element |
|  | CAT-box | GCCACT | cis-acting regulatory element related to meristem expression |

**Table S2 Cis-element prediction of upstream sequence of *GausACLB-2* gene.**
