# Supplementary material for: ATP-citrate lyase B (ACLB) negatively affects cell death and resistance to Verticillium wilt
Source: BMC Plant Biol. 2022 Sep 16;22:443. doi: 10.1186/s12870-022-03834-z (PMC9479425; doi:10.1186/s12870-022-03834-z)
Supplement: Supplementary file 8 — Additional file 8: Table S3. Comparison of homology between ACLB protein in G. australe, G. hirsutum and G. barbadense. [file 12870_2022_3834_MOESM8_ESM.docx]

| Identity (%) | GausACLB-1 | GausACLB-2 | GbACLB-1A | GbACLB-1D | GbACLB-2A | GbACLB-2D | GhACLB-1A | GhACLB-1D | GhACLB-2A | GhACLB-2D |
| --- | --- | --- | --- | --- | --- | --- | --- | --- | --- | --- |
| GausACLB-1 | 608 (100%) | 95% | 52% | 97% | 95% | 95% | 97% | 97% | 95% | 95% |
| GausACLB-2 | 578 | 608 (100%) | 50% | 96% | 99% | 99% | 96% | 95% | 99% | 99% |
| GbACLB-1A | 322 | 310 | 327  (100%) | 52% | 50% | 51% | 52% | 53% | 50% | 51% |
| GbACLB-1D | 594 | 589 | 321 | 608  (100%) | 97% | 96% | 100% | 98% | 97% | 96% |
| GbACLB-2A | 578 | 605 | 309 | 590 | 608  (100%) | 99% | 97% | 95% | 100% | 99% |
| GbACLB-2D | 579 | 605 | 312 | 587 | 603 | 608  (100%) | 96% | 96% | 99% | 99% |
| GhACLB-1A | 594 | 589 | 321 | 608 | 590 | 587 | 608  (100%) | 98% | 97% | 96% |
| GhACLB-1D | 595 | 583 | 326 | 598 | 583 | 584 | 598 | 608  (100%) | 95% | 96% |
| GhACLB-2A | 578 | 605 | 309 | 590 | 583 | 608 | 590 | 583 | 608  (100%) | 99% |
| GhACLB-2D | 580 | 606 | 312 | 588 | 604 | 607 | 588 | 585 | 604 | 608  (100%) |

**Table S3 Comparison of homology between ACLB protein in *G. australe*, *G. hirsutum* and *G. barbadense*.**

MEGA software was used to compare homology between protein sequences.
