# Supplementary material for: ATP-citrate lyase B (ACLB) negatively affects cell death and resistance to Verticillium wilt
Source: BMC Plant Biol. 2022 Sep 16;22:443. doi: 10.1186/s12870-022-03834-z (PMC9479425; doi:10.1186/s12870-022-03834-z)
Supplement: Supplementary file 9 — Additional file 9: Table S4. Primers used in this study. [file 12870_2022_3834_MOESM9_ESM.docx]

**Table S4 Primers used in this study.**

| **Primer name** | | **Sequences (5'-3')** | **Use in this syudy** |
| --- | --- | --- | --- |
| pAtACLB-1F | ATGCTTTTCCTTAAAAAAAA | | Clone *AtACLB-1* |
| pAtACLB-1R | GATAAAACGTCAGGATTATA | |  |
| pAtACLB-2F | ATGGCAACGGGACAGCTTTTT | | Clone *AtACLB-2* |
| pAtACLB-2R | TTACTTGGTGTACAACACAT | |  |
| pGausACLB-1F | ATGGCCACTGGACAGTTATT | | Clone *GausACLB-1* |
| pGausACLB-1R | TCACTTGGTGTAGAGAACAT | |  |
| pGausACLB-2F | ATGGCCACCGGACAGTTGTT | | Clone *GausACLB-2* |
| pGausACLB-2R | TCACTTAGTGTAGAGAACAT | |  |
| pGbACLB-1AF | ATGGAGTCTGCCCAGGGAAA | | Clone *GbACLB-1A* |
| pGbACLB-1AR | TCACTTGGTGTAGAGAACAT | |  |
| pGbACLB-1DF | ATGGCCACTGGACAGTTATT | | Clone *GbACLB-1D* |
| pGbACLB-1DR | TCACTTGGTGTAGAGAACAT | |  |
| pGbACLB-2AF | ATGGCCACCGGACAGTT | | Clone *GbACLB-2A* |
| pGbACLB-2AR | TCACTTAGTGTAAAGAACAT | |  |
| pGbACLB-2DF | ATGGCCACCGGACAGTTGTT | | Clone *GbACLB-2D* |
| pGbACLB-2DR | TCACTTAGTGTAGAGAACAT | |  |
| pGhACLB-1AF | ATGGCCACTGGACAGTTATT | | Clone *GhACLB-1A* |
| pGhACLB-1AR | TCACTTGGTGTAGAGAACAT | |  |
| pGhACLB-1DF | ATGGCCACTGGACAGTTATT | | Clone *GhACLB-1D* |
| pGhACLB-1DR | TCACTTGGTGTAGAGAACAT | |  |
| pGhACLB-2AF | AACAACTGTCCGGTGGCCAT | | Clone *GhACLB-2A* |
| pGhACLB-2AR | TCACTTAGTGTAAAGAACAT | |  |
| pGhACLB-2DF | ATGGCCACCGGACAGTTGTT | | Clone *GhACLB-2D* |
| pGhACLB-2DR | TCACTTAGTGTAGAGAACAT | |  |
| pGausACLB-2proF | TACAAAAATTCTCAAATTTTT | | Clone *GausACLB-2* promoter |
| pGausACLB-3proR | CATGGCTTCGACTCAAAGAGACC | |  |
| VGbACLB-2F | ATTCTGTGAGTAAGGTTACCGAATTCAGATGGAGTCTGCACAGGGA | | For constructing VIGS vector |
| VGbACLB-2R | AGACGCGTGAGCTCGGTACCGGATCCAGAGACACAGGGACCGTGAT | |  |
| VGhACLB-2F | ATTCTGTGAGTAAGGTTACCGAATTCGAACTTGTGCACGCCTCTTC | | For constructing VIGS vector |
| VGhACLB-2R | AGACGCGTGAGCTCGGTACCGGATCCTTCCCAGCTCTTGCAGTCAC | |  |
| qGausACLB-2F | TCCATGTGTCTCTGGTGCTC | | qRT-PCR for *GausACLB-2* |
| qGausACLB-2R | ATAAGGTGTAAGGCCCCGGT | |  |
| qGbACLB-2F | AGATGGAGTCTGCACAGGGA | | qRT-PCR for *GbACLB-2* |
| qGbACLB-2R | AGAGACACAGGGACCGTGAT | |  |
| qGhACLB-2F | GAACTTGTGCACGCCTCTTC | | qRT-PCR for *GhACLB-2* |
| qGhACLB-2R | TTCCCAGCTCTTGCAGTCAC | |  |
| qAtACLB-2F | AAGCTCTTTTTCGGGCAGGA | | qRT-PCR for *AtACLB-2* |
| qAtACLB-2R | CCAACAGTAGCCGGTCCAAT | |  |
| qGhWAKY23F | ACACCAACTCCAGCTGACAC | | qRT-PCR for *GhWAKY23* |
| qGhWAKY23R | TCAAATTTAAGGTTCATCCCACTGT | |  |
| qGhOsl57F | GCTGGAGATAGCGCTGCTTA | | qRT-PCR for *GhOsl57* |
| qGhOsl57R | AGAATGCAGCCATCCGACAT | |  |
| qGhSGRF | TTGCCAGAGTCATGCCAAGAT | | qRT-PCR for *GhSGR* |
| qGhSGRR | AGACGTAAACAAGAAGCAAACACA | |  |
| qGhPR1F | CGTGGGGTTAGTCTTGGTCC | | qRT-PCR for *GhPR1* |
| qGhPR1R | GCTGTAATCGTAGTCGGCCT | |  |
| qGhEDS1-F | GGCAGACCAAGACGCTACAGATACA | | qRT-PCR for *GhEDS1* |
| qGhEDS1-R | GCAGCAACAGCTCCTCTACCTCAA | |  |
| qGhPAD4-F | GGATGGAAGAATGGAAAGAAATGAA | | qRT-PCR for *GhPAD4* |
| qGhPAD4-R | GAACTAGGAAAGCAGACTAAGGAACCA | |  |
| qGhICS1-F | ATCACCTGAATGGAAGGCGTT | | qRT-PCR for *qGhICS1* |
| qGhICS1-R | CCCAAGTCCATGAAAGGGCAT | |  |
| qGhNPR1-F | GCGAATCGTTGCTTTCTTCTTCA | | qRT-PCR for *GhNPR1* |
| qGhNPR1-R | CACGTGGTGCTGTTGTTGTTACTG | |  |
| qGhPR5-F | GCCGTGATTCATACAGTTATCCTCA | | qRT-PCR for *GhPR5* |
| qGhPR5-R | TTGGCTCTTACTTCCGACCATCT | |  |
| qAtEDS1-F | CGAAGACACAGGGCCGTA | | qRT-PCR for *AtEDS1* |
| qATEDS1-R | AAGCATGATCCGCACTCG | |  |
| qAtPAD4-F | GGTTCTGTTCGTCTGATGTTT | | qRT-PCR for *AtPAD4* |
| qAtPAD4-R | GTTCCTCGGTGTTTTGAGTT | |  |
| qAtICS1-F | GCAAGAATCATGTTCCTACC | | qRT-PCR for *AtICS1* |
| qAtICS1-R | AATTATCCTGCTGTTACGAG | |  |
| qAtNPR1-F | GAGACTCTTGCCTCTTAGTGTAATTT | | qRT-PCR for *AtNPR1* |
| qAtNPR1-R | GCACACCTGCAGCAATAATAC | |  |
| qAtPR1-F | GCTCAAGATAGCCCACAAGATTA | | qRT-PCR for *AtPR1* |
| qAtPR1-R | CCTCTTAGTTGTTCTGCGTAGC | |  |
| qAtPR5-F | TGTGTCTCTGACCTCAACGC | | qRT-PCR for *AtPR5* |
| qAtPR5-R | TCCGGTACAAGTGAAGGTGC | |  |
| Y8991F | CGGTGGTGTGAAGAAGCCTCAT | | cotton reference gene |
| Y8991R | AATTTCACGAACAAGCCTCTGGAA | |  |
| qAtUbq5F | GACGCTTCATCTCGTCC | | *Arabidopsis* reference gene |
| qAtUbq5R | CCACAGGTTGCGTTAG | |  |
| ITS1-F | AAAGTTTTAATGGTTCGCTAAGA | | fungal biomass |
| STVe1-R | CTTGGTCATTTAGAGGAAGTAA | |  |
| LP | TGCACAATTGATTAACCACTTTC | | *aclb-*2 verification primer |
| RP | GAAGGCGTTTCACGTCCTGT | |  |
| LB1.3 | TAGCATCTGAATTTCATAACCAATCTCGATACAC | |  |
| OEGausACLB-2F | GAGAACACGGGGGACTCTAGAGGATCCGGCTCTTTCATTTGC | | For constructing pBI121: *GausACLB-2* vector |
| OEGausACLB-2R | TGTTTGAACGATCGGGGAAATTCACAATGCTCGAGGCTCGTAA | |  |
| GFPGausACLB-2F | ATTTACGAACGATAGGGTACGGATCCGGCTCTTTCATTTGC | | For constructing GFP vector |
| GFPGausACLB-2R | GCCCTTGCTCACCATGGATCCTTAGTGTAGAGAACATCCT | |  |
